# Genetic variation in *ZmKW1* contributes to kernel weight and size in dent corn and popcorn

**DOI:** 10.1111/pbi.14279

**Published:** 2024-01-01

**Authors:** Long Zhang, Miaomiao Fu, Wenyu Li, Yongbin Dong, Qiang Zhou, Qilei Wang, Xinyu Li, Jie Gao, Yan Wang, Han Wang, Yayong Li, Jiechen Wang, Yongrui Wu, Yuling Li

**Affiliations:** ^1^ State Key Laboratory of Wheat and Maize Crop Science, Collaborative Innovation Center of Henan Grain Crops, College of Agronomy Henan Agricultural University Zhengzhou China; ^2^ College of Forestry Henan Agricultural University Zhengzhou China; ^3^ National Key Laboratory of Plant Molecular Genetics, CAS Center for Excellence in Molecular Plant Sciences Shanghai Institute of Plant Physiology and Ecology Chinese Academy of Sciences Shanghai China; ^4^ College of Agronomy Xinyang Agricultural and Forestry University Xinyang China

**Keywords:** *Zea mays*, popcorn, kernel weight and size, endosperm cell development, ubiquitination pathway

## Abstract

Kernel weight is a critical factor that essentially affects maize (*Zea mays*) yield. In natural inbred lines, popcorn kernels exhibit overtly smaller sizes compared to dent corn kernels, and kernel weight, which is controlled by multiple genetic loci, varies widely. Here, we characterized a major quantitative trait locus on chromosome 1, responsible for controlling kernel weight (*qKW1*) and size. The *qKW1* locus encodes a protein containing a seven in absentia domain with E3 ubiquitin ligase activity, expressed prominently from the top to the middle region of the endosperm. The presence and function of *qKW1* were confirmed through *ZmKW1* gene editing, where the mutations in *ZmKW1* within dent corn significantly increased kernel weight, consistent with alterations in kernel size, while overexpression of *ZmKW1* had the opposite effect. *ZmKW1* acts as a negative regulator of kernel weight and size by reducing both the number and size of the endosperm cells and impacting endosperm filling. Notably, the popcorn allele *qKW1* ^*N*^ and the dent corn allele *qKW1* ^*D*^ encode identical proteins; however, the differences in promoter activity arise due to the insertion of an Indel‐1346 sequence in the *qKW1* ^*N*^ promoter, resulting in higher expression levels compared to *qKW1* ^*D*^, thus contributing to the variation in kernel weight and size between popcorn and dent corn kernels. Linkage disequilibrium analysis of the 2.8 kb promoter region of *ZmKW1* in a dataset comprising 111 maize association panels identified two distinct haplotypes. Our results provide insight into the mechanisms underlying kernel development and yield regulation in dent corn and popcorn, with a specific focus on the role of the ubiquitination system.

## Introduction

Maize (*Zea mays*) underwent domestication from teosinte approximately 9000 years ago (Kistler *et al*., 2018). Over millennia of artificial selection, maize has been known for its extreme genetic diversity at the population level (Hufford *et al*., 2021), leading to substantial variations in kernel characteristics among different maize inbred lines (Zhou *et al*., 2019). In contrast, popcorn typically has comparatively smaller kernels than other types of corn and is inferior to dent corn in yield and other agronomic traits (Sweley *et al*., 2013). The underlying molecular genetic mechanism responsible for the persistence of small kernels in popcorn remains unknown. Therefore, it holds significant scientific importance to understand the molecular characteristics of genes that affect the weight and size of popcorn kernels, with the aim of advancing genetic improvements in maize yield.

Sustainable food production to meet the demand of a growing global population while minimizing the environmental impact of agricultural expansion is a pressing challenge (Burgess *et al*., 2022). To improve maize yield, it is necessary to understand the molecular mechanisms of kernel weight and size in order to utilize diverse germplasm resources. Kernel weight is a major agronomic trait in maize and is one of the most complex quantitative traits, and many quantitative trait loci (QTLs) with natural variations have been identified (Borrás and Vitantonio‐Mazzini, 2018; Li *et al*., 2023). Despite the identification of many mutations affecting kernel weight and size, our understanding of QTLs and their metabolites responsible for genetic variations in kernel weight and size within natural maize populations remains limited (Li *et al*., 2023).

Diverse populations have been utilized for QTL mapping studies, which uncover many QTLs associated with kernel weight and size, unevenly distributed across all 10 maize chromosomes (Liu *et al*., 2014). Nonetheless, the identification of major QTLs that have consistent effects across populations, generations, and environments would benefit the efficiency of marker‐assisted selection and be valuable for further cloning (Hospital, 2009). Near‐isogenic lines (NILs) and chromosome segment substitution lines (CSSLs) with identical genetic backgrounds to the recurrent parents have gained widespread usage for QTL verification and fine‐mapping in plants (Sasaki *et al*., 2017). One major QTL, *qHKW1*, encodes a CLAVATA1 (CLV1)/BARELY ANY MERISTEM (BAM)‐related receptor kinase‐like protein, which accounts for 18.4% of the kernel weight variation in the Zheng58 × SK recombinant inbred line (RIL) (Yang *et al*., 2019). Another QTL, *Zm00001d048451*/*qKW9*, encodes a DYW subgroup PPR protein that reduces kernel weight by affecting photosynthesis (Huang *et al*., 2020). *ZmEXPB15*, through its interaction with ZmNAC11 and ZmNAC29, promotes nucellus elimination, thereby positively controlling kernel size and weight (Sun *et al*., 2022b). *qHKW3* harbours the gene *Zm00001d044081*, which encodes a homeobox‐leucine zipper protein (ATHB‐4), affecting kernel weight and size by regulating kernel filling (Sun *et al*., 2022a). In addition, several genes controlling maize grain development have been identified, and their biological roles, such as ZmGRAS11 (Ji *et al*., 2022), opaque2 (Zhang *et al*., 2016), Smk11 (Ren *et al*., 2023), *crk2* (Zhou *et al*., 2023), *dek* (Garcia *et al*., 2017), HSP90.6 (Xu *et al*., 2023), and TaDA1 (Liu *et al*., 2020), have been revealed. Hence, it is crucial to uncover the genetic basis of major QTLs or candidate genes controlling kernel size and weight in maize and gain insights into the molecular mechanisms underlying yield‐related traits.

In our previous study, we identified a major QTL associated with 100‐kernel weight (100‐KW) mapped within the bins 1.03–1.04 on chromosome 1 in maize, denoted as *qKW1* (Li *et al*., 2011). *qKW1* showed a consistent effect on 100‐KW across different geographical locations and successive generations. Other studies have also detected a QTL in this genomic region associated with kernel size and weight, indicating that *qKW1* is a main‐effect QTL within stable genetic penetrance in natural populations (Austin and Lee, 1996; Berke and Rocheford, 1995; Guo *et al*., 2008; Melchinger *et al*., 1998; Yang *et al*., 2012a). In this work, we employed a combination of fine mapping and association mapping to narrow down the major QTL for kernel size and weight, *qKW1*, within a 42.1 kb fragment on maize chromosome 1. The gene *ZmKW1* encodes a seven in absentia (SINA) domain protein containing E3 ubiquitin ligase activity. Our analysis, focusing on the largest polymorphism, Indel‐1346, led to the classification of 111 maize‐inbred lines into two distinct haplotypes, *qKW1* ^*N*^ and *qKW1* ^*D*^. Analysis of 512 dent corn accessions revealed that the *qKW1* ^*N*^ and *qKW1* ^*D*^ haplotypes were associated with low and high 100‐KW, respectively. *ZmKW1* regulates grain‐filling, cell size, and cell number within the endosperm in kernels. These results may provide a molecular biological foundation for further research and crop breeding to improve the kernel weight of maize.

## Results

### *qKW1* is a major QTL that controls kernel weight and size

**Figure 1:**
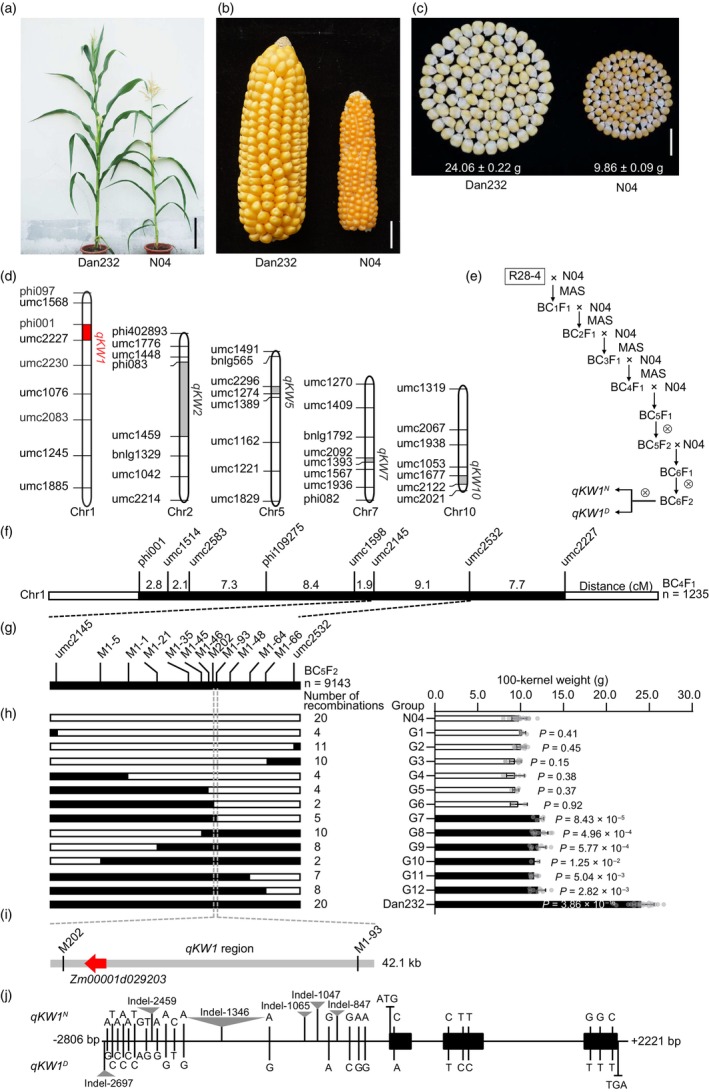
*ZmKW1*, a candidate gene of *qKW1*, regulates kernel weight and size in maize. (a) Dan232 and N04 plants. Scale bar, 20 cm. (b) Dan232 and N04 ears. Scale bar, 2 cm. (c) 100‐kernel weight of Dan232 and N04. Scale bar, 2 cm. (d) QTLs affecting kernel weight in Dan232. (e) Genetic background of near‐isogenic lines for fine mapping of *qKW1*. MAS, Marker‐Assisted Selection. (f) Locations of *qKW1* in the population genetic map of BC_4_F_1_ with 1235 plants. (g) Fine mapping of the *qKW1* region using the 9143 BC_5_F_2_ population. Genotypes of recombinants were assayed by sequencing a 42.1 kb region between M202 and M1‐93. (h) The left panel shows the graphical genotypes of 12 representative recombinants. The white and black segments indicate the regions for N04 and Dan232, respectively. The bar graphs on the right compare the 100‐kernel weight of homozygous recombinants and homozygous nonrecombinants within each recombinant‐derived BC_5_F_2_ population. (i) Progeny testing of the recombinants narrowed down *qKW1* to a 42.1‐kb physical region containing only one annotated gene, *ZmKW1* (*Zm00001d029203*). (j) Natural variations of *ZmKW1* structure and alleles in *qKW1* ^*D*^ and *qKW1* ^*N*^.

To identify the QTLs associated with the small‐kernel weight of popcorn, we previously used a regular dent inbred line Dan232 and a popcorn inbred line N04 to create three populations, RIL, F_2:3_, and BC_2_F_2_ (Li *et al*., 2011). Dan232 showed a higher plant height, longer ear length, and larger kernel size than N04 (Figure 1a,b). The 100‐Kernel Weight (100‐KW) of Dan232 (24.06 ± 0.22 g) was 144.02% higher than that of N04 (9.86 ± 0.09 g) (Figure 1c). Through QTL mapping, we identified at least five loci associated with both kernel size and weight across the three populations, of which the one located within an interval between two molecular markers, phi001 and umc2227, on the short arm of chromosome 1 displayed the greatest effect on kernel size and weight (Figure 1d). Hence, we designated this major QTL as *Kernel Weight 1* (*qKW1*).

R28‐4, derived from 258 F_9_ populations, was a RIL that carried a *qKW1* fragment from Dan232 (*qKW1* ^*D*^) and showed a large kernel size and weight (Li *et al*., 2011). To fine‐map *qKW1*, a series of continuous backcross populations was generated using R28‐4 as the large‐KW parent and N04 as the recurrent backcross parent (Figure 1e). Using the analysis of 1235 BC_4_F_1_ individuals, the QTL was positioned between markers umc2145 and umc2532 (Figure 1f). Subsequently, in BC_5_F_2_, we analysed 75 recombinants between umc2145 and umc2532, and found that individuals with homozygous *qKW1* ^*D*^ and *qKW1* ^*N*^ (N04 allele) showed larger and smaller kernel sizes and weights, respectively, whereas heterozygotes displayed intermediate phenotypes (Figure 1g,h; Figure S1). High‐resolution mapping utilizing 9143 BC_5_F_2_ individuals further narrowed down *qKW1* to a 42.1 kb region marked by M202 and M1‐93, where only one gene, *Zm00001d029203*, encoded a protein containing a seven‐in absentia (SINA) domain with E3 ubiquitin ligase activity, annotated based on Zm‐B73‐REFERENCE‐GRAMENE‐4.0 (Figure 1i; Figure S3b,c). Thus, *Zm00001d029203* was designated as *ZmKW1*. Sequence analysis revealed six Indels (insertions and/or deletions) and 16 SNPs (single nucleotide polymorphisms) within the promoter region between *qKW1* ^*D*^ and *qKW1* ^*N*^ (Figure 1j; Figure S2). Notably, the largest Indel was a 1033 bp fragment located in the *qKW1* ^*N*^ promoter (−1346 bp relative to the start codon), absent in the *qKW1* ^*D*^ promoter. Seven SNPs were also found in the coding sequence, but they did not cause amino acid substitution (Figure 1j; Figure S3a). These results indicated that the sequence variations in the promoter region between *qKW1* ^*D*^ and *qKW1* ^*N*^ contribute to the differences in the kernel phenotypes of *ZmKW1*.

**Figure 2:**
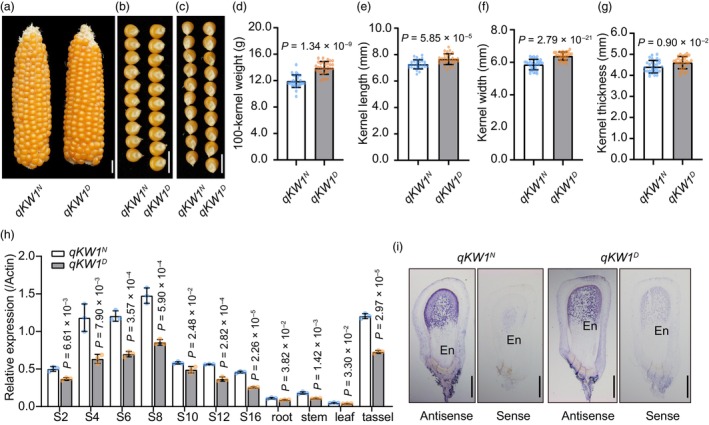
*ZmKW1* negatively regulates kernel weight and size. (a–c) Comparison of ear (a) and kernel (b and c) morphology between *qKW1* ^*N*^ and *qKW1* ^*D*^ plants. Scale bar, 1 cm. (d–g) Phenotype analysis of 100‐kernel weight (d), kernel length (e), kernel width (f) and kernel thickness (g) in *qKW1* ^*N*^ and *qKW1* ^*D*^. *P* values were determined by student's *t*‐tests. (h) Expression pattern of *ZmKW1* in *qKW1* ^*N*^ and *qKW1* ^*D*^. S2‐S16: developing seeds from 2 to 16 DAP (day after pollination). Data are mean ± SD (*n* = 3 technical repeats). (i) RNA *in situ* hybridization of *ZmKW1* in an 8‐DAP kernel. Scale bar, 1 mm.

We selected an individual of G8 type from the BC_5_F_2_ generation and used it for backcrossing to obtain the BC_6_F_1_ generation (Figure 1e,h). By self‐pollinating BC_6_F_1_, we generated a nearly isogenic line (NIL) of *ZmKW1*, *qKW1* ^*D*^, and its isogenic control, *qKW1* ^*N*^, by marker‐assisted selection (Figure 2a). Compared with *qKW1* ^*N*^, *qKW1* ^*D*^ showed a significant increase in 100‐KW, kernel length, kernel width, and kernel thickness (Figure 2b–g; Table S1), while there were no differences observed in ear length, kernel number per row, and kernel row number (Table S1). Quantitative RT‐PCR results revealed that *ZmKW1* was generally expressed across all examined tissues, with the highest transcript abundance detected during the early stages of seed development (4–8 days after pollination, DAP) and in tassels. In all tissues, the transcription levels of *ZmKW1* in *qKW1* ^*N*^ were significantly higher than those in *qKW1* ^*D*^ (Figure 2h). Spatial expression pattern analysis of *ZmKW1* during kernel development showed that *ZmKW1* transcripts were most abundant at 8 DAP (Figure 2h). Additionally, RNA *in situ* hybridization with a *ZmKW1* antisense probe in 8 DAP kernels showed the predominant expression of *ZmKW1* in the endosperm, with the strongest hybridization signal detected from the top to the middle region of the endosperm (Figure 2i). Collectively, these results indicated a negative correlation between the expression level of *ZmKW1* and kernel size and weight.

### *ZmKW1* negatively controls kernel weight and size

**Figure 3:**
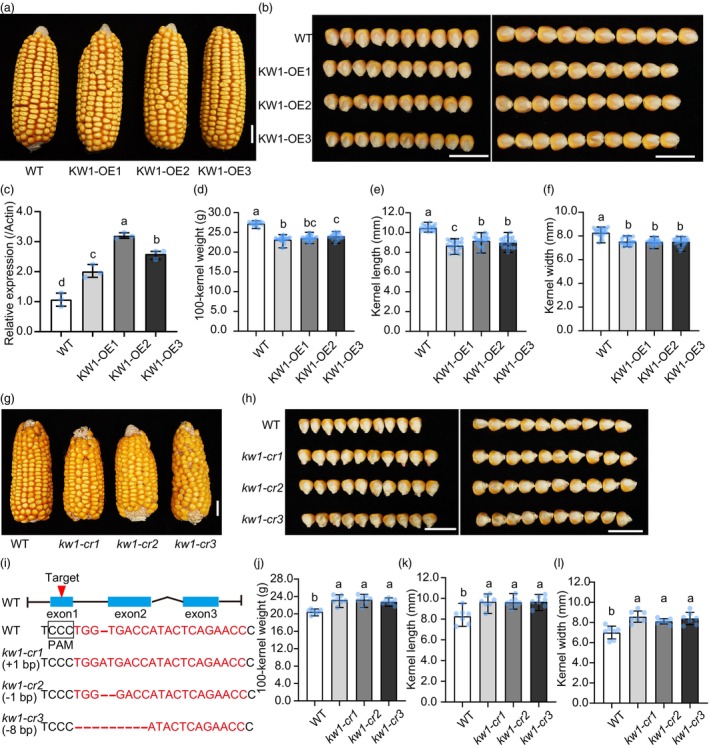
Validation of the *ZmKW1* function by overexpression and CRISPR. (a) Ear morphology of WT and *ZmKW1* overexpression lines (KW1‐OE1, KW1‐OE2 and KW1‐OE3) driven by the *ubiquitin* promoter. Scale bar, 2 cm. (b) Kernel phenotypes of WT and *ZmKW1* overexpression lines. Scale bar, 2 cm. (c) The expression analysis of *ZmKW1* in WT and *ZmKW1* overexpression lines by quantitative RT‐PCR. Data are mean ± SD (*n* = 3 biologically independent samples). Different letters above the column represent statistically significant differences at *P* < 0.05 (one‐way ANOVA, Tukey's honestly significant difference). (d–f) Statistical analysis of 100‐kernel weight (d), kernel length (e) and kernel width (f) of WT and *ZmKW1* overexpression lines. Data are mean ± SD (*n* = 20–22 plants). Different letters above the column represent statistically significant differences at *P* < 0.05 (one‐way ANOVA, Tukey's honestly significant difference). (g) Ear morphology of WT and *zmkw1* mutants. Scale bar, 2 cm. (h) Kernel phenotypes of WT and *zmkw1* mutants. Scale bar, 2 cm. (i) Schematic representation of CRISPR/Cas9‐edited sequences in *ZmKW1* alleles. The *kw1*‐cr1, *kw1*‐cr2 and *kw1*‐cr3 mutants contain an “A” insertion, “T” deletion and “TGGTGACC” deletion, respectively. (j–l) Analysis of 100‐kernel weight (j), kernel length (k) and kernel width (l) of WT and *ZmKW1* mutants. Data are mean ± SD (*n* = 7 plants). Different letters above the column represent statistically significant differences at *P* < 0.05 (one‐way ANOVA, Tukey's honestly significant difference).

To verify the function of *ZmKW1* in kernel weight regulation, we employed maize *ubiquitin* promoter‐driven overexpression (*Ubi::ZmKW1*) in B104. Three representative overexpression (OE) lines (KW1‐OE1, KW1‐OE2, and KW1‐OE3) were generated (Figure 3a,b). Quantitative RT‐PCR analysis revealed that the transcript levels of *ZmKW1* were 2‐ to 3‐fold higher in the OE lines compared to wild‐type (WT) (Figure 3c). The 100‐KW, kernel length, kernel width, and kernel thickness of the OE lines were significantly reduced when compared to those of WT (Figure 3d–f). Furthermore, we utilized the clustered regularly interspaced short palindromic repeats (CRISPR)/CRISPR‐associated protein 9 (Cas9) technology to generate *zmkw1* null mutants (*kw1‐cr1*, *kw1‐cr2*, and *kw1‐cr3*) in KN5585 background, which bore specific genetic modifications, including an A insertion, a T deletion, and an 8 bp deletion, respectively (Figure 3g–i). In contrast to the OE lines, *zmkw1‐cr* mutants showed a significant increase in 100‐KW and kernel size compared to the WT (Figure 3j–l).

We obtained another mutant, *ZmKW1*, from the Maize Genetics and Genomics Database (https://www.maizegdb.org/uniformmu). Through mutant screening, we identified three alleles that bore a *Mu* element insertion in the promoter region of *ZmKW1* (*kw1‐mu1*, *kw1‐mu2*, and *kw1‐mu3*) in W22 (Figure S4a,b). The transcript levels of *ZmKW1* in these three *Mu*‐insertion mutants were lower than those of the WT (Figure S4c). Similar to the *kw1‐cr* mutants, these mutants showed a significant increase in both 100‐KW and kernel size compared to the WT (Figure S4d–f). Notably, there was a direct correlation observed between the expression level of *ZmKW1* and the extent to which the kernel weight and size were increased in these mutants (Figure S4g–i). Together, these results confirm that *ZmKW1* functions as a negative regulator of kernel weight and size in maize.

### Indel‐1346 confers enhanced *ZmKW1* promoter activity

**Figure 4:**
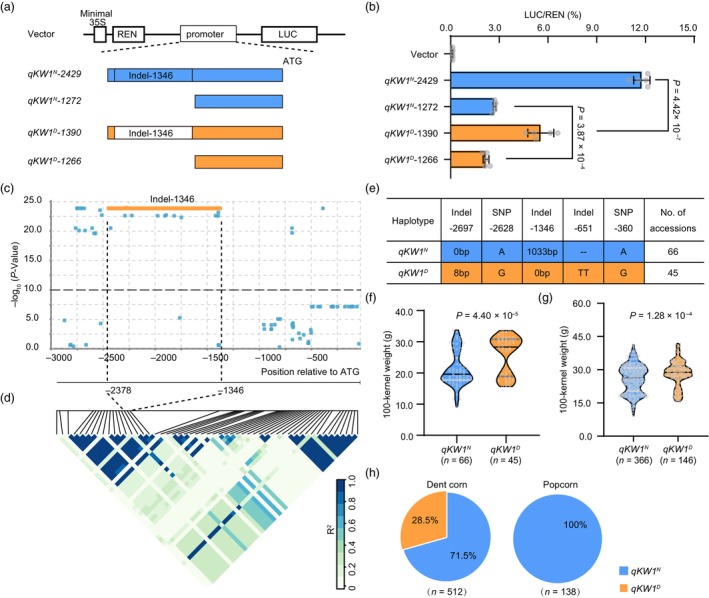
The Indel‐1346 in the *ZmKW1* promoter contributes to kernel weight in maize. (a) Schematic diagram illustrating the constructs used in the transient transcriptional activity assays. The luciferase (*LUC*) gene, driven by the 2.8 kb promoter sequence from *qKW1* ^*N*^ or *qKW1* ^*D*^, was used as the reporter gene to assess the effect of Indel‐1346. (b) Evaluation of LUC gene expression drive by the promoter from *qKW1* ^*N*^ or *qKW1* ^*D*^ in maize endosperm. Data are mean ± SD (*n* = 5 biological replicates); *P* values were determined by the Student's *t*‐test. (c) Association analysis of sequence variations in the *ZmKW1* 2.8 kb promoter among diverse maize inbred lines. (d) Linkage disequilibrium analysis of the 2.8 kb *ZmKW1* promoter in a maize association panel of 111 accessions. (e) Haplotype analysis of the *ZmKW1* promoter. (f) Distribution of 100‐kernel weight for haplotype *qKW1* ^*N*^ (*n* = 66 accessions) and haplotype *qKW1* ^*D*^ (*n* = 45 accessions). (g) Allele effects on 100‐kernel weight in a panel of 512 diverse dent corns. (h) Proportions of the N04 and Dan232 alleles and their co‐occurrence within dent corn and popcorn germplasm groups. Data from 512 and 138 accessions diversity maize inbred accessions were integrated.

Sequence analysis of the promoter region between *qKW1* ^*D*^ and *qKW1* ^*N*^ revealed the presence of six Indels (insertions and/or deletions) (Figure 1j; Figure S2), where the largest Indel was a 1033 bp fragment located at −1346 bp relative to the start codon in the *qKW1* ^*N*^ promoter, while this fragment was absent from the *qKW1* ^*D*^ promoter. To determine whether these sequence polymorphisms in the *ZmKW1* promoter underlie differential expression levels, transient expression assays were performed in maize endosperms. As the largest polymorphism, we amplified promoter fragments from each allele using two forward primers located upstream and downstream of the Indel, along with a single reverse primer located upstream of the start codon (Figure 4a). This led to the generation of four effector constructs: *qKW1* ^*N*^ *‐2429*, *qKW1* ^*N*^‐1272, *qKW1* ^*D*^‐1390, and *qKW1* ^*D*^‐1266, corresponding to the promoter fragments of *qKW1* ^*N*^ (−2429 and −1272 bp) and *qKW1* ^*D*^ (−1390 and −1266 bp) fused with the *luciferase* (*LUC*) reporter (Figure 4a). Notably, *qKW1* ^*N*^‐2429 displayed the highest LUC activity, which was about 3.36‐, 1.12‐, and 4.40‐fold higher than that of *qKW1* ^*N*^‐1272, *qKW1* ^*D*^‐1390, and *qKW1* ^*D*^‐1266, respectively. The LUC activity of *qKW1* ^*D*^‐1390 was over twice that of *qKW1* ^*D*^‐1266 (Figure 4b). While the LUC activity of *qKW1* ^*N*^‐1272 was significantly greater than that of *qKW1* ^*D*^‐1266, the causal polymorphisms responsible for the greatest differences in promoter activities are likely to be located within the largest Indel‐1346.

To investigate the potential associated between the largest Indel‐1346 in the *ZmKW1* promoter and kernel weight in natural maize inbred lines, a promoter region approximately 2.8 kb was amplified and sequenced from a panel of 111 diverse maize inbred lines (Figure 4c,d; Table S3). Through this analysis, we characterized a total of 82 genetic variants, including 51 SNPs and 31 Indels, among which 5 variants showed a significant association with kernel weight after accounting for multiple testing with Bonferroni correction (Figure 4c,d; Table S2). Based on the presence or absence of the Indel‐1346, the 111 inbred lines could be classified into two distinct haplotypes: 66 inbred lines belonged to the *qKW1* ^*N*^ haplotype, and 45 inbred lines belonged to the *qKW1* ^*D*^ haplotype (Figure 4e; Table S3). Notably, the 100‐KW of the *qKW1* ^*N*^ haplotype inbred lines was significantly lower than that of the *qKW1* ^*D*^ (Figure 4f; Table S3). Further analysis conducted on a diverse panel comprising 512 dent inbred lines revealed that the *qKW1* ^*N*^ and *qKW1* ^*D*^ haplotypes were associated with low and high 100‐KW, respectively (Figure 4g; Table S4). Among the 512 dent inbred lines, 71.5% belonged to the *qKW1* ^*N*^ haplotype, while 138 popcorn inbred lines were all *qKW1* ^*N*^ haplotype, suggesting the *qKW1* ^*N*^ allele has been subjected to artificial selection during popcorn breeding (Figure 4h; Tables S4 and S5).

### *ZmKW1* affects cell development and grain‐filling

**Figure 5:**
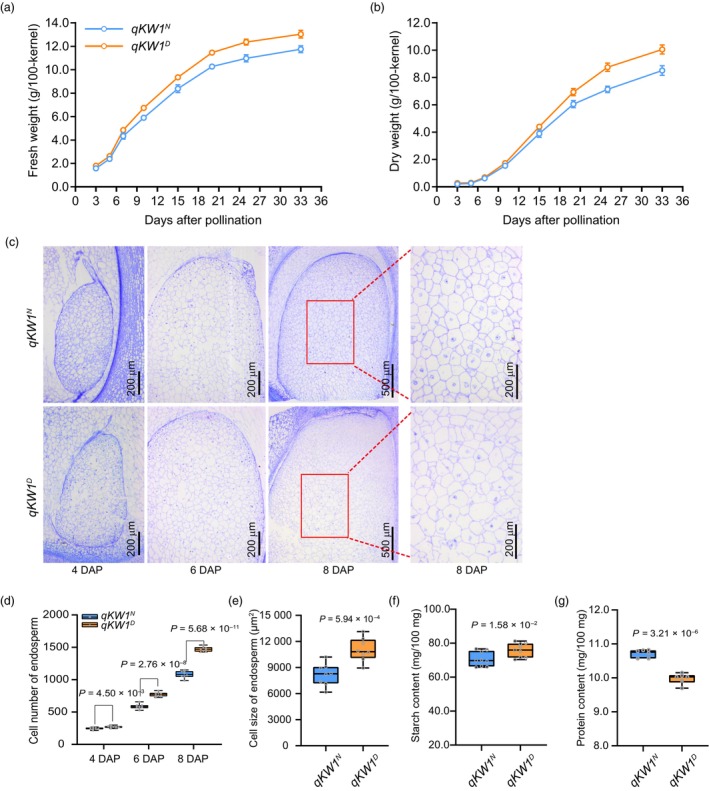
*ZmKW1* participates in endosperm development. (a, b) Fresh weight (a) and dry weight (b) measurements of kernels at 3–33 DAP kernel during grain‐filling in *qKW1* ^*N*^ and *qKW1* ^*D*^. (c) Light microscopy images of semi‐thin sections of developing endosperms from 4 to 8 DAP in *qKW1* ^*N*^ and *qKW1* ^*D*^. (d, e) Cell number (d) and size (e) in 8 DAP endosperm of *qKW1* ^*N*^ and *qKW1* ^*D*^. Data are mean ± sd *P* values were determined by Student's *t*‐tests. (f, g) Total starch contents (f) and total protein contents (g) in *qKW1* ^*N*^ and *qKW1* ^*D*^ kernels. Data are means ± SE, *n* = 3 biological replicates.

To infer the function of *ZmKW1* in regulating kernel size and weight, we determined the fresh and dry weights of developing kernels. At 3 and 5 DAP, both *qKW1* ^*N*^ and *qKW1* ^*D*^ kernels showed similar fresh weight, and by 7 DAP, the fresh weight of *qKW1* ^*D*^ kernels was evidently higher than that of *qKW1* ^*N*^. In the grain‐filling stage at 10 DAP, there was a progressive increase in the disparity of fresh weight between *qKW1* ^*D*^ and *qKW1* ^*N*^ (Figure 5a). The dry weight showed a similar trend to the fresh weight, but the disparity in dry weight lagged behind that of fresh weight variation and was evident after 15 DAP (Figure 5b). Histological analysis of *qKW1* ^*N*^ and *qKW1* ^*D*^ kernels from 2 to 20 DAP revealed differences in their endosperm development (Figure S5a). Measurement of endosperm size from 2 to 20 DAP consistently showed that *qKW1* ^*N*^ endosperm was consistently smaller than *qKW1* ^*D*^ endosperm (Figure 5c; Figure S5b). Additionally, we observed the cell number and size at 4 to 8 DAP and found that the cell number of *qKW1* ^*N*^ endosperm was less than that of *qKW1* ^*D*^ endosperm (Figure 5d), and the cell size of *qKW1* ^*D*^ endosperm was significantly smaller than that of *qKW1* ^*N*^ endosperm (Figure 5e). Observation of KW1‐OE2 and *kw1‐cr2* endosperm cells of 6 DAP kernels showed that both the cell number and size decreased in KW1‐OE2 compared to the WT, while the cell number and size increased in *zmkw1‐cr2* (Figure S7a,b). These results suggested that *ZmKW1* regulates kernel weight and size by affecting both the number and size of the endosperm cells during grain‐filling.

The content of starch and total protein (including zein and non‐zein) in mature dried kernels was determined, and significant differences were observed between *qKW1* ^*N*^ and *qKW1* ^*D*^, transgenic lines and their corresponding WT (Figure 5f,g; Figures S6 and S7). Compared to *qKW1* ^*N*^, *qKW1* ^*D*^ kernels showed a 7.5% increase in starch content and a 6.8% decrease in protein content; the change in zein content was significant (*P* = 1.74 × 10^−5^), while the content of non‐zein was relatively unchanged (Figure 5f,g; Figure S6). Compared to the corresponding WT, the starch content in KW1‐OE2 and *kw1‐cr2* decreased by 4.8% and increased by 6.8%, respectively; the protein content in KW1‐OE2 and *kw1‐cr2* increased by 8.2% and decreased by 8.7%, respectively (Figure S7). Taken together, these results suggest that *ZmKW1* has a negative regulatory effect on kernel weight and size by reducing the number and size of cells in the endosperm, as well as affecting endosperm filling.

### ZmKW1 encodes a SINA protein with E3 ubiquitin ligase activity

ZmKW1 is predicted to encode a protein containing a SINA domain. To investigate its potential functional classification, we conducted a search within the National Center for Biotechnology Information (NCBI) and the Institute for Genomic Research (TIGR) databases. Based on the obtained information, we constructed a phylogenetic tree in which these proteins were annotated as E3 ubiquitin protein ligases (Figure S8). Further analysis involved generating functional domain profiles for four selected SINA protein sequences (Figure S9). Alignment of the deduced amino acid sequences of ZmKW1 with seven representative SINA proteins revealed the presence of conserved domains, including the RING finger, SIAH, and TRAF domains, although they exhibit variability in their N‐terminal regions (Figures S3 and S9). The amino acid sequence homology between ZmKW1, OsDIS1, SINAT3, and SINAT4 ranges from 65% to 86% (Figure S9).

**Figure 6:**
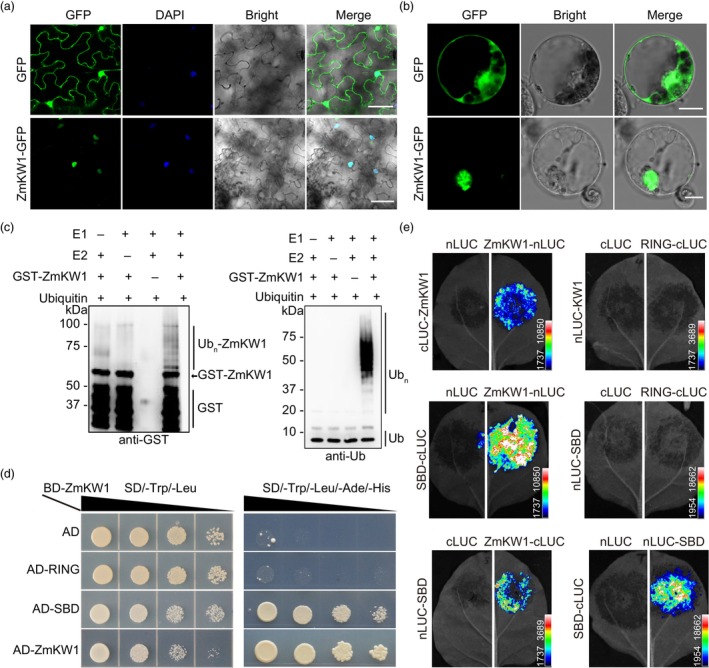
ZmKW1 is a nucleus protein with E3 ubiquitin ligase activity. (a) Subcellular localization of *35S*::GFP and *35S*::ZmKW1‐GFP fusion in tobacco leaf epidermal cells. DAPI was used as a nucleus marker. Scale bar, 50 μm. (b) Transient expression of *35S*::GFP and *35S*::ZmKW1‐GFP fusion proteins in maize protoplasts. Scale bar, 20 μm. (c) E3 activity of the GST‐ZmKW1 fusion protein in the presence of E1, E2, and ubiquitin. The detection of ubiquitinated proteins by protein blot analysis using an anti‐Ub to ubiquitin. (d) Yeast two‐hybrid assay indicates that the SBD domain of ZmKW1 interacts with ZmKW1. (e) LUC assay shows that the SBD domain interacts with ZmKW1 in tobacco leaf cells.

We fused the full‐length ZmKW1 protein (322 amino acids) to the N‐terminus of green fluorescent protein (GFP). Transient expression of the ZmKW1‐GFP fusion construct in tobacco (*Nicotiana benthamiana*) epidermal cells and maize leaf protoplasts showed that ZmKW1 localized to the nucleus (Figure 6a,b), which is similar to the subcellular localization patterns reported for SINAT3 in *Arabidopsis* and OsDIS1 in rice (Ning *et al*., 2011; Xia *et al*., 2020b). Previous studies have demonstrated that many plant SINA proteins encode functional E3 ubiquitin ligases (Wang *et al*., 2018b; Xie *et al*., 2002). To determine whether ZmKW1 also functions as an E3 ubiquitin ligase, we generated a fusion protein called ZmKW1 with a glutathione S‐transferase (GST) tag and purified the tagged protein using GST affinity beads. In the presence of ubiquitin, rabbit E1, and human E2 (UBch5b), we observed ubiquitylation of GST‐ZmKW1, evident by the signals detected with GST antibody and Ub antibody (Figure 6c). In contrast, purified GST protein and combinations lacking E1 or E2 showed no ubiquitination signal even after an extended incubation period. These results provide evidence that ZmKW1 is a functional E3 ubiquitin ligase with autonomously undergoing ubiquitination.

To further determine how this circuit was regulated, we tested the ZmKW1 interaction by dimerization (Xia *et al*., 2020a). The ZmKW1 protein was divided into distinct domains, including the RING‐finger domain, SIAH domain, and TRAF domain from the N‐terminus (Figure S3). We performed yeast two‐hybrid assays (Figure 6d) and LUC assays in tobacco leaf epidermal cells (Figure 6e) to assess protein interactions with truncated ZmKW1 protein fragments according to specific domains. The results showed that ZmKW1 did not interact with the RING‐finger domain but rather interacted with the substrate‐binding domain (SBD), that is, contained both SIAH and TRAF domains. Therefore, it is speculated that ZmKW1 undergoes self‐ubiquitination, and its structure contains an SBD domain, which enables the formation of dimers and self‐degradation by self‐ubiquitylation.

### Ubiquitin‐modified ZmKW1 proteome analysis

**Figure 7:**
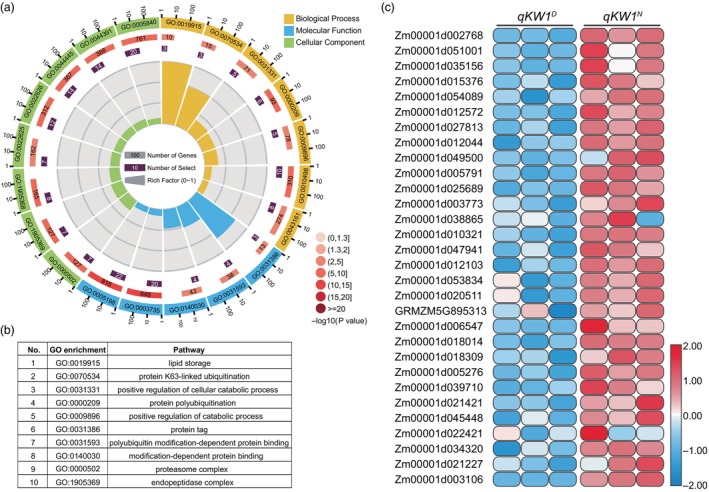
The role of ZmKW1 in maize kernel. (a) GO term enrichment analysis of DEPs in 8 DAP kernels of *qKW1* ^*N*^ and *qKW1* ^*D*^. The circular graph displays four layers, from the outermost to the innermost. The first layer represents the enrichment classification, with the gene number indicated outside the circle. Each colour represents a different category. The second layer shows the number of genes and *P* values for each classification in the background genes. Longer bars indicate more genes, while a redder colour indicates smaller *P* values. The third layer represents the total number of differentially expressed target genes. The fourth layer represents the richness factor values for each classification, with each small bar on the background auxiliary line equivalent to 0.1. DEPs are differentially expressed proteins. (b) Top ten significantly enriched GO terms. (c) According to the intensity ratio of ubiquitinated‐specific enriched peptides, the significantly different proteins were analysed.

To explore the involvement of ZmKW1's E3 ubiquitin ligase in kernel development, we conducted a ubiquitin‐modified proteome profiling analysis using proteins extracted from 8 DAP endosperms of *qKW1* ^*N*^ and *qKW1* ^*D*^ kernels (Table S6). From this analysis, we identified 587 differentially expressed ubiquitinated proteins, 874 ubiquitinated peptides, and 977 ubiquitinated sites (Table S6). We performed enrichment analysis of Kyoto Encyclopedia of Genes and Genomes (KEGG) pathways in the ubiquitinome dataset. We observed preferential enrichment for KEGG pathways related to glycolysis/gluconeogensis (*P* = 0.004) and carbon fixation in biological organisms (*P* = 0.014), among the differentially expressed proteins in *qKW1* ^*N*^ and *qKW1* ^*D*^ (Table S6). A significance analysis of gene ontology (GO) terms was performed to annotate the identified ubiquitinated proteins based on their association with the ribosome, ubiquitin, and protein catabolism. The ubiquitylated GO shared by *qKW1* ^*N*^ and *qKW1* ^*D*^ was screened out, and GO is related to synthesis and degradation of substances related to grain‐filling processes such as starch, protein, and lipid (Dai *et al*., 2021). The identified GO terms were shared, and the background number of GO was between 100 and 1000 (Figure 7a,b; Table S6). Our enrichment analysis of GO terms indicated that the ubiquitinated proteins identified in maize kernels participate in a variety of biological processes and exhibit diverse molecular functions. To identify significantly differentially abundant proteins, we calculated the intensity ratio of peptides specifically enriched for ubiquitination, resulting in the identification of 25 proteins with significant differences (Figure 7c; Table S7). Some of these proteins, with significant differences in ubiquitination levels, had physiological functions during seed development, and the phenotypes of seed size, weight, starch content, and protein content were to varying degrees similar to those of ZmKW1 kernels. From this, we hypothesized that these proteins may be involved in the downstream ubiquitination process of ZmKW1.

## Discussion

In our study, we successfully narrowed down the *qKW1* locus, a major QTL responsible for kernel weight, to a 42.1 kb region, which harbours only one candidate gene, *ZmKW1*. *ZmKW1* encoded an E3 ubiquitin ligase with both a RING‐finger domain and a SINA domain localized to the nucleus. The introgression of the *qKW1* ^*D*^ allele, achieved through marker‐assisted selection from the Dan232 into N04, resulted in increases in kernel length, width, and thickness, as a consequence leading to enhanced 100‐KW.

Previous studies have shown that RING‐finger proteins participate in grain development and affect grain size. For example, in rice, the *GW2* gene, which encodes a RING‐finger type E3 ubiquitin ligase, has been identified as a QTL associated with grain width and weight (Song *et al*., 2007). The WY3 allele of GW2 carries a truncated version of the protein with a 310 amino acid deletion. The presence of the *GW2* allele in WY3 leads to an increase in the number of cells in the spikelet hull, which in turn accelerates the grain milk‐filling rate, resulting in an increase in grain width, weight, and yield (Song *et al*., 2007). GW2 negatively regulates cell division by targeting its substrate(s) for regulated proteolysis with proteasomes. Additionally, homologues of rice *GW2* have been reported in both wheat and maize (Li *et al*., 2010; Yang *et al*., 2012b). The expression pattern of *TaGW2‐A* is characterized by high levels during the grain cell division stage and late grain‐filling, in contrast to the roles of rice *GW2* and *qKW1*. TaGW2‐A has been identified as a positive regulator of grain size in rice. In maize, two homologous genes of rice *GW2*, *ZmGW2‐CHR4* and *ZmGW2‐CHR5*, have been reported, with each located on chromosomes 4 and 5, respectively (Li *et al*., 2010). In wheat, TaSDIR1‐4A is a member of the SDIR1 family with E3 ubiquitin ligase activity, and studies involving gene silencing in wheat and transgenic *Arabidopsis* plants have indicated that TaSDIR1‐4A negatively regulates grain size. This discrepancy in regulation may be attributed to the role of TaERF3 (ethylene response factor) as a transcriptional repressor of TaSDIR1‐4A in Hap‐4A‐2 (Wang *et al*., 2020; Yan *et al*., 2022). In addition, in *Arabidopsis*, two RING‐type E3 ubiquitin ligases, DA2 and BB/EOD1, have been identified. BB and EOD1 are functional alleles, and both DA2 and BB/EOD1 are negatively correlated with seed size in *Arabidopsis* (Disch *et al*., 2006; Xia *et al*., 2013). Another protein with a RING‐finger and wd40‐associated ubiquitin‐like (RAWUL) domain, Gnp4/LAX2, regulates grain length by affecting cell expansion (Zhang *et al*., 2018).

The fact that the protein sequences encoded by *qKW1* ^*N*^ and *qKW1* ^*D*^ do not differ suggests that there are significant differences in the transcript levels associated with the kernel phenotype during kernel development. The LUC activity of the truncated *qKW1* ^*N*^ (−2429 and −1272 bp) and *qKW1* ^*D*^ (−1390 and −1266 bp) promoter fragments was analysed (Figure 4a). It was observed that the LUC activity of the *qKW1* ^*N*^‐2429 promoter fragment was significantly stronger than that of the others, which provided convincing evidence that the activity induced by Indel‐1346 in the promoter region is responsible for the kernel weight and size variation (Figure 4b). Indel‐1346, therefore, represents the largest causal polymorphism in promoter activity. Insertions or deletions of Indels quantitatively affect promoter activity, consequently influencing *ZmKW1* transcript levels. *ZmKW1* is a negative regulator of kernel weight and size. The deletion of Indel‐1346 in the *qKW1* ^*D*^ promoter reduces the function of *qKW1* ^*D*^, resulting in a significant increase in kernel length, width, thickness, and 100‐KW in *qKW1* ^*D*^ relative to *qKW1* ^*N*^. Reduced expression of *ZmKW1* results in increased kernel weight and size, whereas overexpression decreases kernel weight and size. Given the generally small‐kernel character of popcorn compared to that of regular maize, we analysed the promoters from 111 different maize accessions through linkage disequilibrium. We classified them into two haplotypes, *qKW1* ^*D*^ and *qKW1* ^*N*^, according to Indel‐1346 (Figure 4). Further analysis, integrating data from 512 dent corn accessions, revealed that the kernel weight of the *qKW1* ^*N*^ haplotype was generally lower than that of *qKW1* ^*D*^, suggesting that the *qKW1* ^*N*^ allele has undergone artificial selection in popcorn breeding.

The analysis of the ubiquitin‐modified proteome profiling revealed significant differences in the abundance of 25 selected proteins (Figure 7c; Table S7). Among these proteins, Oleosin (Zm00001d002768) contributes to high lipid accumulation in seeds (Ha *et al*., 2019; Yu *et al*., 2021). Glyceraldehyde‐3‐phosphate dehydrogenase 2 (Zm00001d035156) and glyceraldehyde‐3‐phosphate dehydrogenase 3 (Zm00001d051001) have been shown to modulate carbon fluxes, seed number, and seed oil accumulation (Guo *et al*., 2014; Rius *et al*., 2008). Phosphoglycerate kinase 3 (Zm00001d015376), phospholipid transfer protein 30 (Zm00001d012572), pyruvate, and orthophosphate dikinase 2‐like (Zm00001d010321) affect storage protein accumulation, starch biosynthesis, and metabolic regulation (Lappe *et al*., 2018; Zhang *et al*., 2016). Moreover, differential proteins of 40S ribosomal proteins (Zm00001d027813, Zm00001d035094, Zm00001d035312, Zm00001d007810, Zm00001d049500, and Zm00001d045448) and 60S ribosomal proteins (Zm00001d038865) are associated with kernel size and starch granules in maize (Qi *et al*., 2016; Wang *et al*., 2018a). The significantly different proteins also included small ubiquitin‐related modifier 1b (Zm00001d012044), putative ubiquitin family protein (Zm00001d053834), and ubiquitin fusion protein/polyubiquitin 2 (Zm00001d020511), which directly participate in the ubiquitination process and affect seed length and underfilled endosperm (Pei *et al*., 2019; Zhang *et al*., 2021). Yeast two‐hybrid screening of ZmKW1 interaction proteins revealed frequent interactions with E3 ubiquitin‐protein ligase SINAT4, suggesting that ZmKW1 may ubiquitinate other E3 ubiquitin ligases and indirectly impact downstream target proteins (Table S8). In addition, regulatory particle non‐ATPase (Zm00001d047941), Histone H4/H2A (Zm00001d005791/Zm00001d006547), polyA binding protein (Zm00001d005276/Zm00001d003106), and 26S protease regulatory subunit (Zm00001d047941/Zm00001d018409) have been reported to play regulatory roles in seeds (Book *et al*., 2009; Kurepa *et al*., 2009; Tiwari *et al*., 2008; Yang *et al*., 2016).

The principle underlying ubiquitin label‐free quantitative proteomics involves the specific enrichment of ubiquitinated peptides from samples by high‐affinity lysine antibodies. Proteins with significant differences, as described above, were identified through protein abundance analysis. It is assumed that downstream ubiquitinated target proteins have been nearly or completely degraded at 8 DAP in ZmKW1 kernels, rendering their abundance undetectable. Proteins with undetectable abundance in *qKW1* ^*N*^ but high abundance in *qKW1* ^*D*^ could potentially represent the differential proteins in this assumption. Notably, the MAP3K epsilon protein kinase 1 (Zm00001d001978) and SURF1‐like protein (Zm00001d005712) showed the largest differences in abundance (Table S9). Previous studies have reported the *YDA* gene, which encodes a mitogen‐activated protein kinase kinase kinase (MAPKKK), with the regulation of seed size via the *AN3‐YDA* gene cascade (Meng *et al*., 2017). Mutations in SURFEIT1 (SURF1), the siliques from heterozygous plants, show abnormal seeds, embryos from which look somewhat retarded in development and abnormal seeds shrank and acquired a dark‐brown colour upon maturation (Gras *et al*., 2020). These proteins, mentioned above, have been reported to be associated with seed development and display significant differences in the ubiquitination proteome. Thus, it is highly likely that they play functional roles in the ubiquitination process of ZmKW1.

While it is crucial to gain a comprehensive understanding of kernel development to improve grain yields through genetic manipulation, our knowledge regarding the genetic mechanisms that determine the final kernel size and weight in crops remains limited. *ZmKW1* represents a key regulator of maize kernel weight and size, offering promising opportunities for breeding efforts aimed at improving the yield of staple grain crops. Taken together, the genetic variation and molecular mechanisms underlying the different alleles in dent corn and popcorn, as demonstrated in our study, provide valuable insights for the molecular breeding of kernel weight and size in maize.

## Methods

### Fine mapping of *qKW1*

N04, a popcorn inbred line, was selected from the Chinese popcorn BL03 by our research group (Figure 1a–c). Dan 232, a normal dent inbred line, was bred from Lu 9 Kuan × Dan340 (Figure 1a–c). The F_1_ generation was obtained by crossing Dan232 as the male parent and N04 as the female parent. In our previous study (Li *et al*., 2011), we mapped a major QTL controlling kernel weight, named *qKW1*, to the interval between SSR markers phi001 and umc2227 on chromosome 1 using a population of recombinant inbred lines (RILs) derived from the cross N04 × Dan232 (Figure 1d). The consistent detection and interpretation of *qKW1* for phenotypic variation in different environments was 20.50%–24.60%. All primer sequences used in this study are listed in Table S10.

A fine mapping population was obtained by backcrossing N04. Using 1235 BC_4_F_1_ individuals, we mapped the *qKW1* locus to an interval between two markers, umc2145 and umc2532, on chromosome 1 (Figure 1f). These two markers were genetically supported by the recombinant events of 18 individuals. To further narrow down the location, we created a BC_5_F_2_ population through backcrossing with N04. 20 Indel markers were used for fine mapping, and we obtained 75 recombinant individual plants, among which *qKW1* was located within the 42.1 kb region between M202 and M1‐93, which only contained one annotated gene, *ZmKW1* (*Zm00001d029203*) (Figure 1g–i). To obtain homozygous *qKW1* ^*N*^ and *qKW1* ^*D*^, BC_5_F_2_ independent ears with a high kernel weight were planted in two groups (G6 and G7 in Figure 1h) and 15 plants from each group were backcrossed with N04, yielding 30 BC_6_F_2_ ear (Figure 1e). Then, 20 plants from each BC_6_F_2_ ears were self‐pollinated, yielding 600 BC_6_F_3_ ears that were measured for 100‐kernel weight. Individual ears with a uniformly high kernel weight in each subgroup were identified as *qKW1* ^*D*^, while ears with a kernel weight similar to N04 were designated *qKW1* ^*N*^ (Figure 1e). *qKW1* ^*N*^ and *qKW1* ^*D*^ were propagated through self‐pollination.

All maize plants were grown in the fields in the Chinese cities of Zhengzhou (34.9°N, 113.6°E), Shanghai (30.5°N, 121.1°E), or Sanya (18.2°N, 109.3°E). To minimize the effect of environmental factors, we phenotyped and genotyped kernel weight and size at these three sites. Kernels selected from the middle region of ears in a uniform kernel set were used for photography and kernel trait measurement, and at least 100 kernels were measured for each ear. The kernel weight, length, and width were measured using an image analysis method provided with SC‐E software (Wanshen Detection Technology, Hangzhou, China). The kernel thickness was measured using an electronic digital display vernier calliper with fully filled kernels.

### Genetic confirmation

We obtained overexpression and knockout transgenic maize mutants via *Agrobacterium tumefaciens*‐mediated transformation by WiMi Biotechnology (http://www.wimibio.com/) in Jiangsu, China. For the overexpression lines, we amplified the full‐length of *ZmKW1* and inserted it into a vector driven by a ubiquitin promoter (Tian *et al*., 2019). The construct was subsequently introduced into *A. tumefaciens* EHA105 and transformed into B104. Following the standard transformation protocol, three positive events were recovered. For the knockout mutants, we searched for single guide RNA (sgRNA) sites in *ZmKW1* using the website http://cbi.hzau.edu.cn/CRISPR2/ and selected the highest On‐score. A sgRNA targeting exon 1 (+90 to +109) was cloned into the *Psi*I and *Xba*I sites between the maize U6 promoter and U6 terminator (Ji *et al*., 2022). The constructs were introduced into *A. tumefaciens* EHA105 and transformed into KN5585. To identify Cas9‐free transgenic plants with homozygous mutations, we utilized PCR product sequencing and hygromycin selection. The primers used for construction and genotyping are listed in Table S10.

### Associative analysis

The 512 dent maize inbred lines (Table S5) and 138 popcorn inbred lines (Table S4) used for the association analysis were supported by Yongrui Wu (CAS Center for Excellence in Molecular Plant Sciences, Shanghai), Hongjian Zheng (Shanghai Academy of Agricultural Sciences), and our laboratory. A 2800 bp fragment of the *ZmKW1* promoter was sequenced and associated with kernel weight in a natural population of 111 inbred lines (Table S3) from our laboratory. Sequence alignment was performed using Geneious Primer software (https://www.geneious.com/), and association analysis was conducted using the Linear Mixed Model (MLM) from Tassel software. To identify the promoter alleles, a molecular marker Indel‐1346F/R was developed. The statistical genotypes of the 512 dent maize inbred lines and 138 popcorn inbred lines were tested by agarose gel electrophoresis.

### RNA preparation and qRT‐PCR analysis

Total RNA was extracted from roots, stems, leaves, tassels, and seeds using Trizol reagent (Invitrogen, Los Angeles, CA). Fresh tissues were collected in 2 mL RNAse‐free tubes and suspended in 0.3 mL RNA extraction buffer. The homogenized samples were extracted twice, using 0.3 mL phenol–chloroform (pH 4.2) and 0.3 mL chloroform, respectively. The RNA was then extracted using a 1 mL Trizol reagent. The extracted total RNA was further purified by the RNeasy Mini Kit with DNaseI digestion (Qiagen, Dusseldorf, Germany). Equal amounts of RNA were selected for reverse transcription from each sample with the ImProm‐IITM Reverse Transcription System (TAKARA, Shiga, Japan). qRT‐PCR was performed with SYBR Green I (Yeasen, Shanghai, China). The relative expression was calculated using the $2^{-\Delta \Delta C_{t}}$ method, with the maize *Actin* gene as a reference.

### Histocytochemical analysis

For histocytochemical analysis, kernel slices with a thickness of 1 mm were placed onto adhesive slides. Kernels were fixed in FAA buffer (50% ethanol:formaldehyde:acetic acid = 90:5:5 [v/v/v]) and embedded in resin after dehydration through an ethanol gradient for the paraffin section. The semi‐thin sections were fixed in FAA buffer (formaldehyde:acetic acid:ethanol:water = 10:5:50:35 [v/v/v/v]) and then embedded in epoxide resin for semi‐thin sectioning. All sections were stained with 0.1% toluidine blue solution and then photographed in bright field with a Leica DM2500 microscope (Leica, Heidelberg, Germany). Images were taken, and the cell size and number of endosperm cells were measured using ImageJ 1.52a software.

### RNA *in situ* hybridization

The material used for *in situ* hybridization was embedded in paraffin according to the methods described previously (Zhang *et al*., 2015). *In situ* hybridization was conducted using 10 DAP kernels from *qKW1* ^*N*^ and *qKW1* ^*D*^. The *ZmKW1* fragment was amplified by PCR and inserted into the pSPT18 vector. Sense and antisense RNA probes were synthesized *in vitro* using T7 and SP6 RNA polymerase with DIG RNA Labeling Mixture (Roche, Basel, Switzerland). Fresh tissues were obtained and fixed in a 4% paraformaldehyde solution containing 0.1% Triton X‐100 and 0.1% Tween 20 in PBS (Sangon Biotech, Shanghai, China). Tissue processing and *in situ* hybridization experiments were carried out on 10 μm sections, and the sections were observed and imaged with an optical stereomicroscope (M165 FC; Leica, Heidelberg, Germany).

### Determination of protein and starch

The total protein content was determined using a Rapid N EXCEED instrument (elementar, Frankfurt, Germany; Huang *et al*., 2022). Mature seeds, after removing the pericarp and embryo, were dried at 42 °C for at least 12 h and ground into a fine powder using a tissue grinder. For protein determination, 50 mg of the powder was weighed and wrapped in special tin foil as a test sample. The protein content was determined using the Kjeldahl method (Huang *et al*., 2022). 100 mg of powder was used to determine the content of zein and non‐zein. 100 mg of power was incubated with 1 mL of zein protein extraction buffer (70% ethanol, 2% 2‐mercaptoethanol [v/v], 3.75 mM sodium borate, pH 10, and 0.3% SDS) in 2 mL tubes at room temperature. For non‐zein protein extraction, the resulting precipitate was further extracted twice with zein solution buffer and vortexed with 1 mL of non‐zein extraction buffer (12.5 mM sodium borate, 2% 2‐mercaptoethanol [v/v], and 5% SDS) for 2 h at room temperature. Zein and non‐zein were extracted three times and mixed for determination by a BCA protein assay kit (Pierce). The total protein content of the seeds was calculated from the average seed weight. The total starch content was determined with the Total Starch Assay Kit (K‐TSTA; Megazyme, Bray, Ireland) according to the manufacturer's procedure. All measurements were replicated at least three times.

### Subcellular localization

The amplified ZmKW1 coding sequence was inserted into a pCAMBIA1301 plasmid driven by a 35S promoter. The constructed vector was transformed into *Agrobacterium tumefaciens* GV3101 (Weidibio, Shanghai, China) and infiltrated into tobacco leaves. The transfected leaves were observed with an SP8 confocal microscope (Leica, Heidelberg, Germany). We also observed the localization of ZmKW1 in the maize leaf protoplast system. The constructed vectors were extracted using the NucleoBond Xtra Midi (MN, Munich, Germany), according to the manufacturer's instructions, to obtain a concentration over 1 μg/μL. Protoplasts were isolated from maize leaves that had been cultured in the dark for about 3 weeks.

### Protein purification and enzyme activity determination

To generate the GST‐tag fusion protein of ZmKW1, the full‐length CDS of ZmKW1 was amplified and cloned into a PGEX‐4 T‐1 vector using *EcoR*I and *Sal*I enzyme sites (TransGen Biotech, Beijing, China). The constructed vector was then transformed into a Transetta (DE3) Chemically Competent Cell (TransGen Biotech) for protein expression. Following induction with 0.5 mM IPTG, protein purification was performed with the GST 4FF Sefinose Resin Kit (Sangon Biotech, Shanghai, China). The purified protein was concentrated via ultrafiltration and quantified using BSA as a standard. To verify the ubiquitase activity of ZmKW1, a system was set up consisting of 500 ng of purified GST‐ZmKW1, 10 μg of recombinant Ub (Sigma, Darmstadt, Germany), 0.1 μg of rabbit E1 (Boston Biochemicals, Boston, MA), 0.2 μg of E2 UbcH5b (Boston Biochemicals), 2 mm ATP, 50 mm Tris–HCl (pH 7.4), 5 mm MgCl_2_, and 2 mM DTT. After incubation at 30 °C for 2 h, the reaction was terminated by adding 2× SDS‐PAGE loading buffer at 95 °C for 5 min. Ubiquitinated proteins were detected by Western blotting using anti‐GST (Abclonal, Wuhan, China) and anti‐Ubiquitin (Abclonal) antibodies. The protein was detected with GST (1:5000) and Ubiquitin (1:2000) at 4 °C overnight, followed by secondary anti‐mouse‐HRP at a concentration of 1:5000 (Abclonal). Imaging was done with a Tanon‐5200 system (Tanon, Hangzhou, China).

### Protein interaction experiments

For the Bimolecular Luciferase Complementation experiment, the RING domain, SBD domain, and full‐length CDS of ZmKW1 were cloned into JW771 (nLUC) and JW772 (cLUC) vectors (Chen *et al*., 2008). The constructed vectors were then transfected into tobacco leaves via *Agrobacterium tumefaciens*. After 72 h of growth under a 16 h light and 8 h dark cycle, the leaves were injected with 0.8 mM luciferin (Promega, Madison, WI, USA), and the resulting luciferase signals were captured with the Tanon‐5200 system (Tanon, Hangzhou, China). For the yeast two‐hybrid assay, the amplified RING domain, SBD domain, and full‐length CDS of ZmKW1 were inserted into pGBKT7 and pGADT7 vectors. Different combinations of the constructed pGBKT7 and pGADT7 vectors were co‐transformed into the Y2HGold yeast strain (Weidibio).

### Assessment of the ubiquitin‐modified proteome

Label‐free quantitative proteomics was used to study the differences in ubiquitination between *qKW1* ^*D*^ and *qKW1* ^*N*^ on 6 DAP kernels by Shanghai APTBIO Biotech. Each group contained three biological replicates. The 6 DAP kernels were ground to a fine powder using liquid nitrogen and vortexed in UA buffer (8 m Urea, 100 mm Tris/HCl, pH 8.5). DTT was added to the samples to achieve a final concentration of 10 mm. After mixing at 37 °C for 1.5 h, IAA was added, and the reaction was carried out for 30 min in the dark. The concentration was adjusted to 2 m with Tris HCl (pH 8.0). Trypsin was added overnight at 37 °C, and TFA with a final concentration of 0.1% and pH ≤3 was added. After freeze‐drying, IAP was added to redissolve the pretreated beads, which were incubated at 4 °C for 1.5 h. The beads were washed three times with 1 mL of pre‐cooled IAP buffer and water individually. TFA was added and incubated for 10 min. After centrifugation, the supernatant was desalted with C18 STAGE tips. The prepared samples were fractionated by HPLC. Buffer A consisted of 0.1% formic acid in an aqueous solution, while buffer B consisted of 0.1% formic acid in an acetonitrile aqueous solution. The samples were added onto a loading column (Thermo Scientific Acclaim PepMap100, nanoViper C18; Thermo fisher Scientific, Waltham, MA, USA) via an automatic sampler and passed through the analytical column (Thermo Scientific EASY column, C18‐A2; Thermo fisher Scientific) at a flow rate of 300 μL/min. The samples were separated by HPLC and analysed by a Q‐exactive mass spectrometer. A label‐free quantitative proteomics strategy was employed to specifically enrich ubiquitinated peptides from complex samples digested by proteases using high‐affinity antibodies for ubiquitinated lysine in combination with LC–MS/MS analysis. The mass errors for all identified ubiquitinated peptides were predominantly distributed within 10 ppm.

### Statistical analysis

Association analysis was performed with Fisher's exact test, which is powerful in analysing qualitative traits such as kernel weight. The data are presented as the mean ± standard deviation (SD). Statistical analyses were carried out using GraphPad 8.0.2 and Microsoft Excel 2019 software. The significance of differences among different groups was assessed by a one‐way ANOVA with a Tukey's test and a two‐sided Student's *t*‐test. A *P*‐value of less than 0.05 was considered statistically significant. Detailed statistical information can be found in the figure legends.

## Conflict of interest

The authors declare no competing interests.

## Funding

This work was supported by the National Natural Science Foundation of China (31771812, 31971962 and 32272129 to YL), Zhongyuan Scholars in Henan Province (22400510003 to YL), Major Public Welfare Projects of Henan Province (201300111100 to YL), Tackle Program of Agricultural Seed in Henan Province (2022010201 to YL), Technical System of Maize Industry of Henan Province (HARS‐22‐02‐S to YL) and Key Scientific Research Projects for Higher Education of Henan Province (19zx001 to YL).

## Author contributions

Y.L., Y.W., L.Z., M.F., and Q.Z. conceived and designed the experiments. L.Z., M.F., Y.D., Q.Z., Q.W., X.L., J.G., Y.W., H.W., and Y.L. performed experiments. L.Z., M.F., W.L., and Q.Z. analysed data. L.Z., M.F., W.L., Y.W., and Y.L. wrote the manuscript. All the authors edited and proofed the manuscript.

## Supporting information


**Figure S1** Allelic effects of *qKW1* in the maize‐popcorn BC_5_F_2_ population.
**Figure S2** Comparison of the 2.8 kb promoter sequence of *ZmKW1* between *qKW1* ^*N*^ and *qKW1* ^*D*^.
**Figure S3** Comparison of the coding region sequences of the two *ZmKW1* alleles.
**Figure S4** Comparison of phenotypes between T‐DNA mutants and the wild‐type (W22).
**Figure S5** Observation of kernel paraffin sections at different developmental stages.
**Figure S6** Zein and non‐zein protein contents of the endosperm.
**Figure S7** Kernel phenotypes of *ZmKW1* overexpression and knockout lines.
**Figure S8**. Phylogenetic tree analysis of the ZmKW1 protein containing the SINA domain.
**Figure S9** Sequence alignment and domain structure analysis of SINA proteins.

**Table S1** Phenotypes of agronomic traits in *qKW1* ^*N*^ and *qKW1* ^*D*^.
**Table S2** Significant association variants within the 2.8 kb sequenced region around the *ZmKW1* promoter after Bonferroni multiple test correction.
**Table S3** Linkage disequilibrium analysis of the *ZmKW1* promoter in a maize association panel consisting of 111 inbred lines.
**Table S4** Zm*KW1* genotypes in a collection of 138 popcorn inbred lines.
**Table S5** *ZmKW1* genotypes in a collection of 512 dent corn inbred lines.
**Table S6** Ubiquitinated peptides in *qKW1* ^*N*^ and *qKW1* ^*D*^ kernels ubiquitinated proteomics.
**Table S7** Differential proteins in *qKW1* ^*N*^ and *qKW1* ^*D*^ kernel ubiquitination proteomics.
**Table S8** Screening of ZmKW1 interaction proteins by a yeast two‐hybrid library.
**Table S9** Presence–absence proteins in *qKW1* ^*N*^ and *qKW1* ^*D*^ kernel ubiquitination proteomics.
**Table S10** Primers used in this study.

## Data Availability

Data supporting the findings of this work are available within the paper and its source data. The genetic materials generated and analysed in this current study are available from the corresponding author upon request.
