# Supplementary material for: Genetic variation in ZmKW1 contributes to kernel weight and size in dent corn and popcorn
Source: Plant Biotechnol J. 2024 Jan 1;22(6):1453–67. doi: 10.1111/pbi.14279 (PMC11123423; doi:10.1111/pbi.14279)
Supplement: Supplementary file 1 — Figure S1 Allelic effects of qKW1 in the maize‐popcorn BC5F2 population. Figure S2 Comparison of the 2.8 kb promoter sequence of ZmKW1 between qKW1 N and qKW1 D . Figure S3 Comparison of the coding region sequences of the two ZmKW1 alleles. Figure S4 Comparison of phenotypes between T‐DNA mutants and the wild‐type (W22). Figure S5 Observation of kernel paraffin sections at different developmental stages. Figure S6 Zein and non‐zein protein contents of the endosperm. Figure S7 Kernel phenotypes of ZmKW1 overexpression and knockout lines. Figure S8. Phylogenetic tree analysis of the ZmKW1 protein containing the SINA domain. Figure S9 Sequence alignment and domain structure analysis of SINA proteins. [file PBI-22-1453-s002.docx]

**Supplemental Figures**


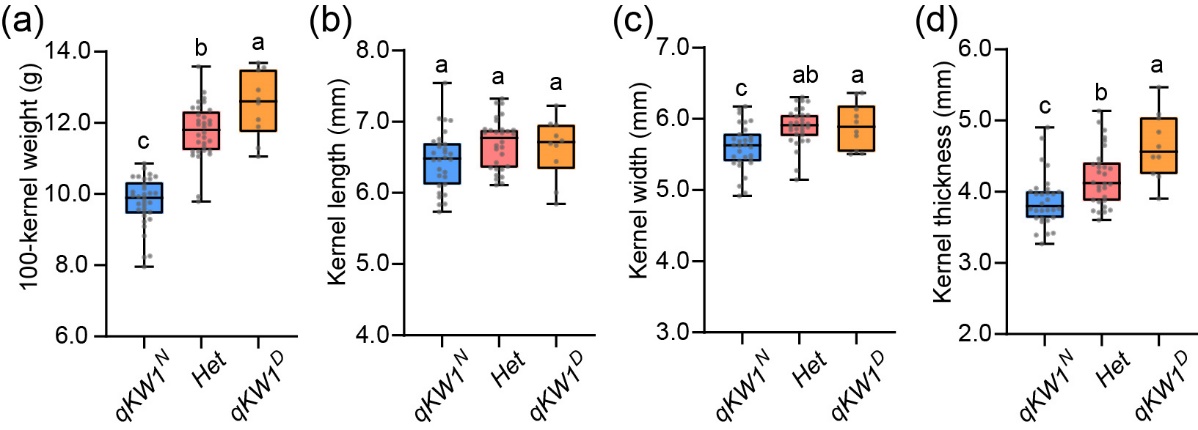


**Figure S1 Allelic effects of *qKW1* in the maize-popcorn BC_5_F_2_ population.** (a-d) Phenotypes of 100-kernel weight (a), kernel length (b), kernel width (c) and kernel thickness (d) in the RIL population. Blue, red and yellow boxes represent RILs that are homozygous for *qKW1^N^*, heterozygous and homozygous for *qKW1^D^*, respectively. Different letters above the column represent statistically significant difference at *P* < 0.05 (one-way ANOVA, Tukey’s honestly significant difference).

**
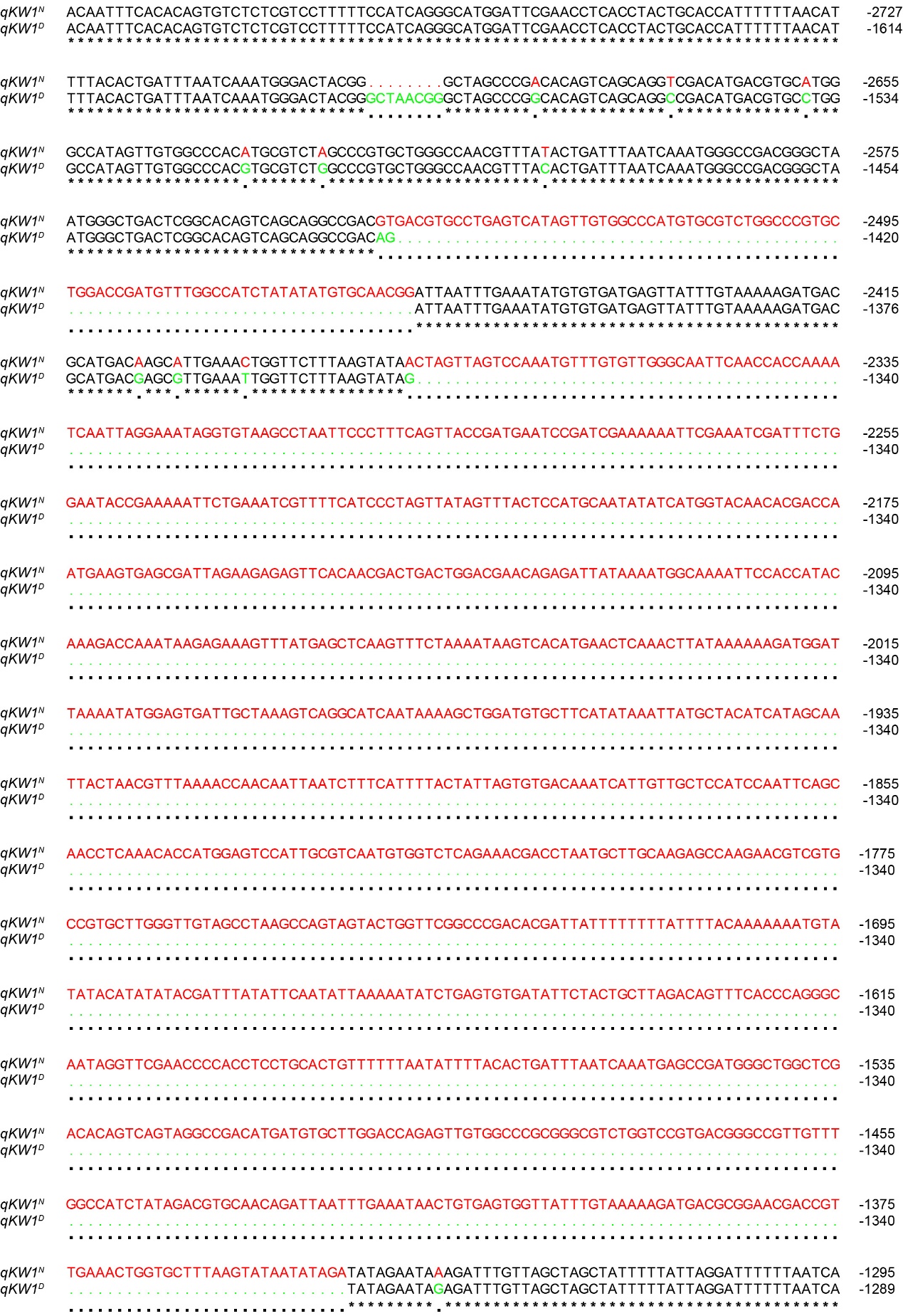
**

**
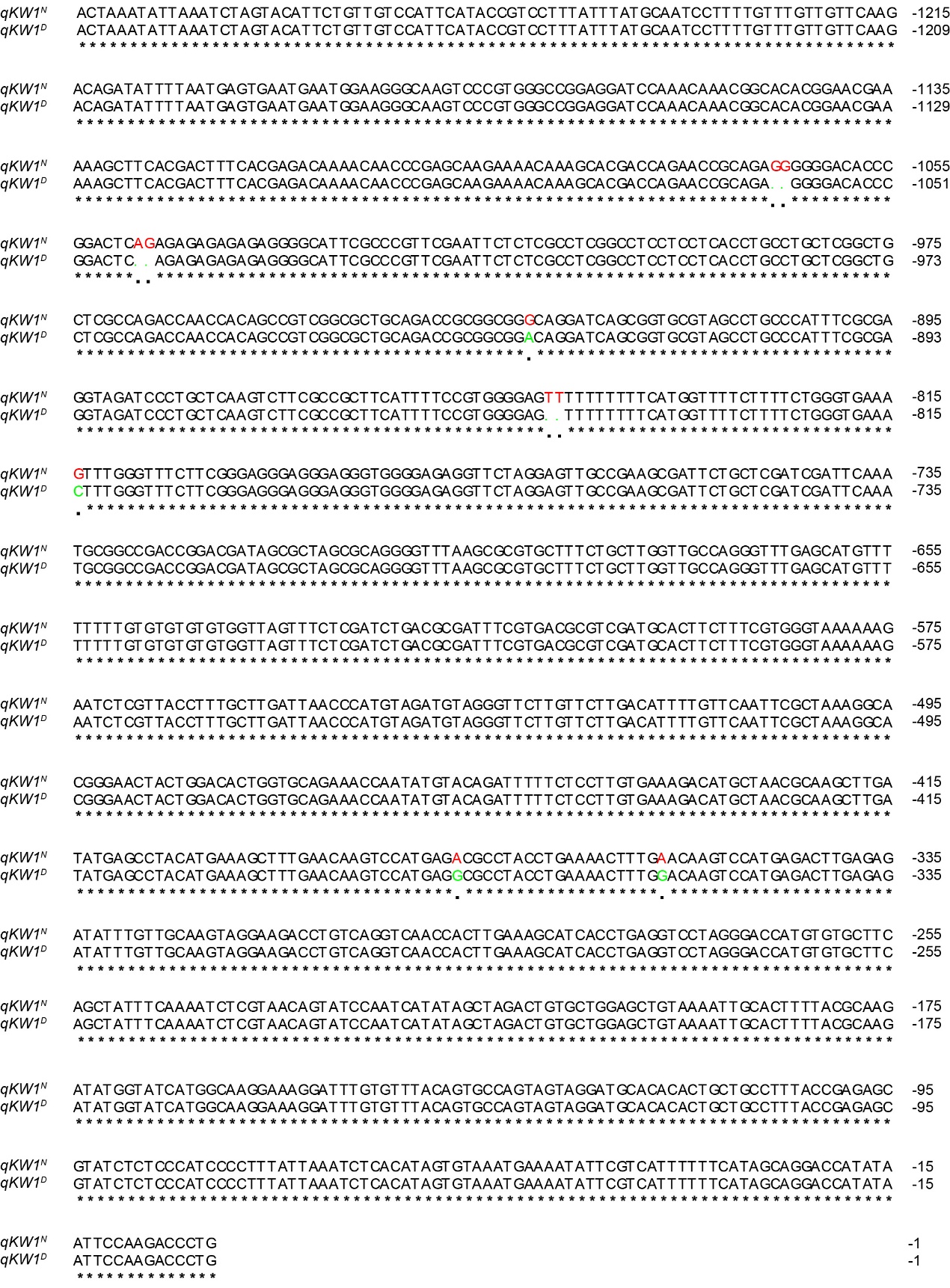
**

**Figure S2. Comparison of the 2.8 kb promoter sequence of *ZmKW1* between *qKW1^N^* and *qKW1^D^*.** Nucleotides relative to the translational start site (ATG) are numbered. The variant SNPs and Indels are highlighted in red and green in *qKW1^N^* and *qKW1^D^*, respectively.

**
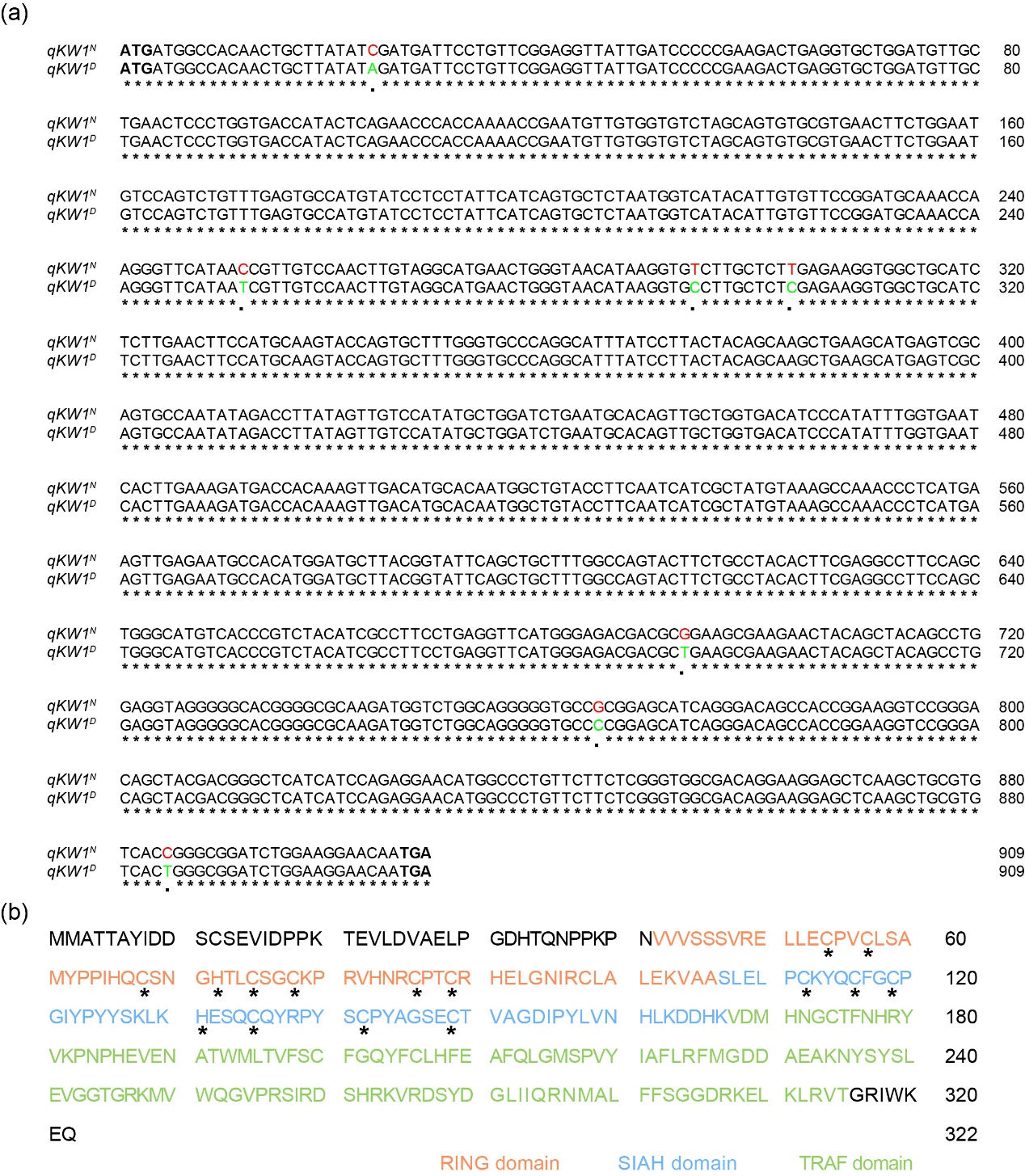
**

**Figure S3. Comparison of the coding region sequences of the two *ZmKW1* alleles.** (a) Sequence variations were identified in the ORF regions of *ZmKW1* between *qKW1^N^* and *qKW1^D^*. The nucleotide variants are indicate in red and green, respectively. (b) The predicted sequence of ZmKW1 protein indicates the presence of RING domain (orange), SIAH domain (blue), and TRAF domain (green).

**
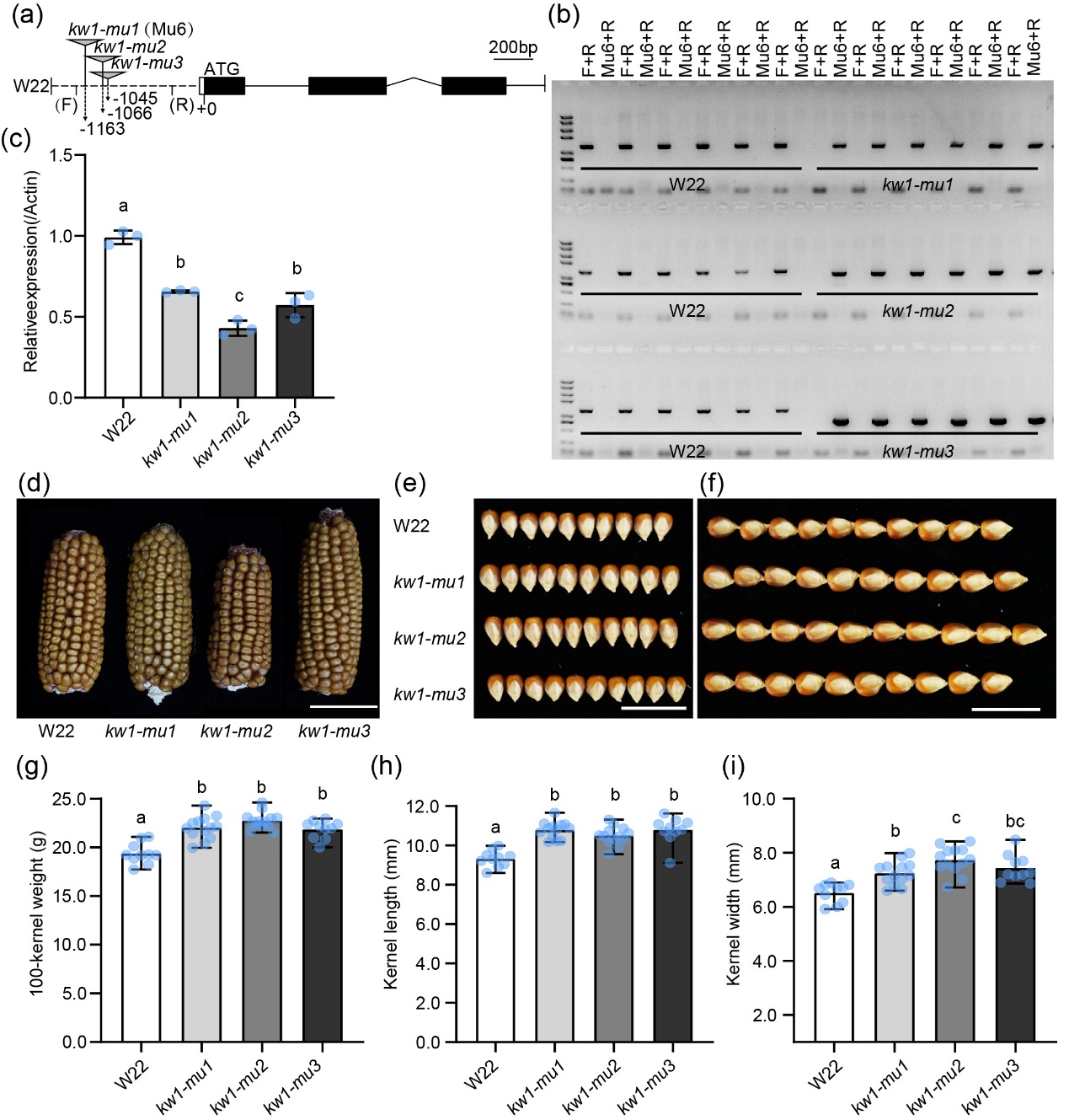
**

**Figure S4. Comparison of phenotypes between T-DNA mutants and the wild-type (W22).** (a) Schematic representation of the *Mu* insertion mutants in the *ZmKW1* promoter. Inverted triangles indicate the *Mu* insertion sites relative to ATG, including -1163 bp (*kw1-mu1*), -1066 bp (*kw1-mu2*) and -1045 bp (*kw1-mu3*). The nucleotide sequences are numbered +1 relative to the first nucleotide of the translation start codon. (b) Identification of the *Mu* insertion mutants. The F and R primers are *ZmKW1-*specific, whereas Mu6 is specific for *Mu* insertion. The primer pairs F + Mu6 and Mu6 + R, but not F + R, produced the correct band from homozygous plants. (c) qRT-PCR analysis of *ZmKW1* expression in 8 DAP endosperm of W22 and its mutants. The *Actin* gene was used as an internal control. Data are presented as mean ± SD for n = 3 biologically independent samples*.* Different letters above the column represent statistically significant difference at *P* < 0.05 (one-way ANOVA, Tukey’s honestly significant difference). (d) The ear phenotypes of WT and the mutants. Scale bar, 50 mm. (e-f) The kernel phenotypes of WT and the mutants. Scale bar, 20 mm. (g-i) 100 kernel weight (g), kernel length (h) and kernel width (i) of WT and the mutants. Data are mean ± SD, Different letters above the column represent statistically significant difference at *P* < 0.05 (one-way ANOVA, Tukey’s honestly significant difference).

**
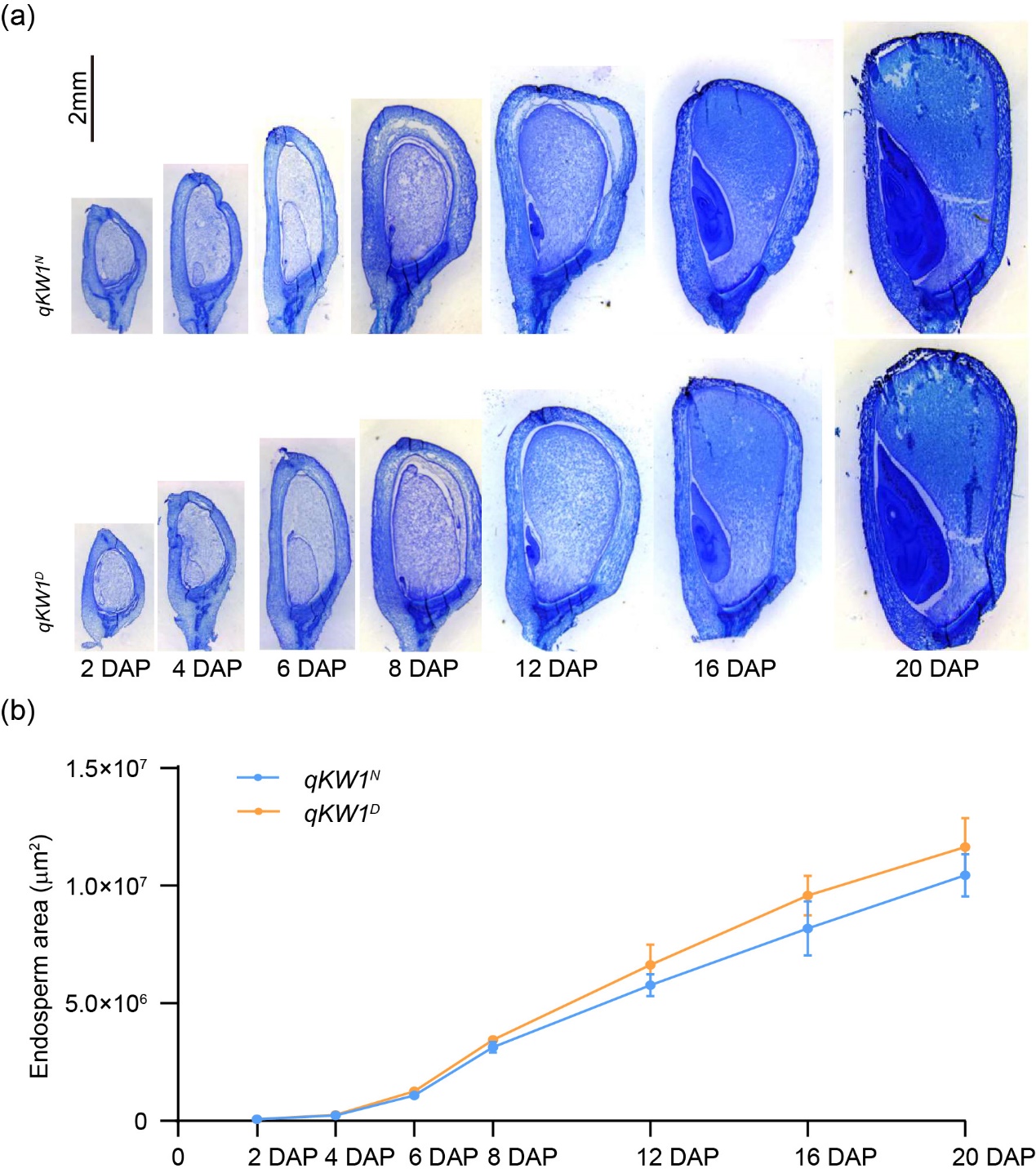
**

**Figure S5. Observation of kernel paraffin sections at different developmental stages.** (a) Longitudinal sections of 2, 4, 6, 8, 12, 16, and 20 DAP from *qKW1^N^* and *qKW1^D^* were stained with toluidine blue, and the development of endosperm and cell division and expansion were observed under a paraffin section microscope. Scale bar, 2 mm. (b) The mean endosperm area of developing *qKW1^N^* and *qKW1^D^* kernels. Data are presented as mean ± SD, n = 8 biologically independent samples.

**
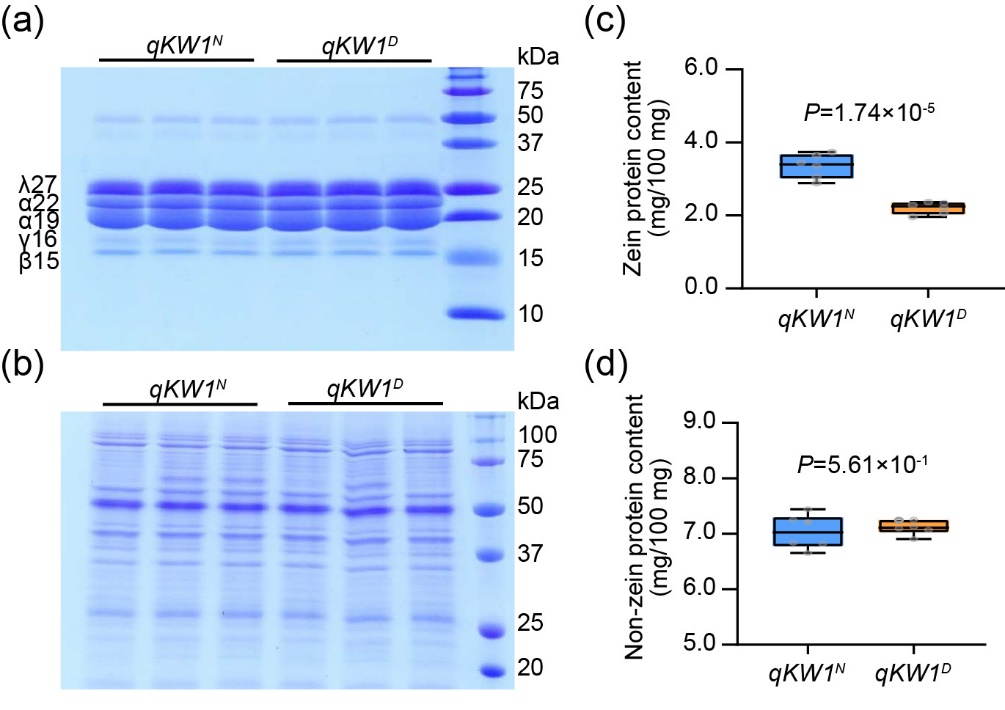
**

**Figure S6.** **Zein and non-zein protein contents of the endosperm.** (a-b) Determination of protein content in *qKW1^N^* and *qKW1^D^* kernels. SDS-PAGE analysis of zein (a) and non-zein (b) from *qKW1^N^* and *qKW1^D^* mature kernel endosperm. (c-d) Zein content (c) and non-zein content (d) of *qKW1^N^* and *qKW1^D^* kernels. Data are means ± SE, n = 3 biological replicates. *P* values were determined by Student’s *t*-test*.*


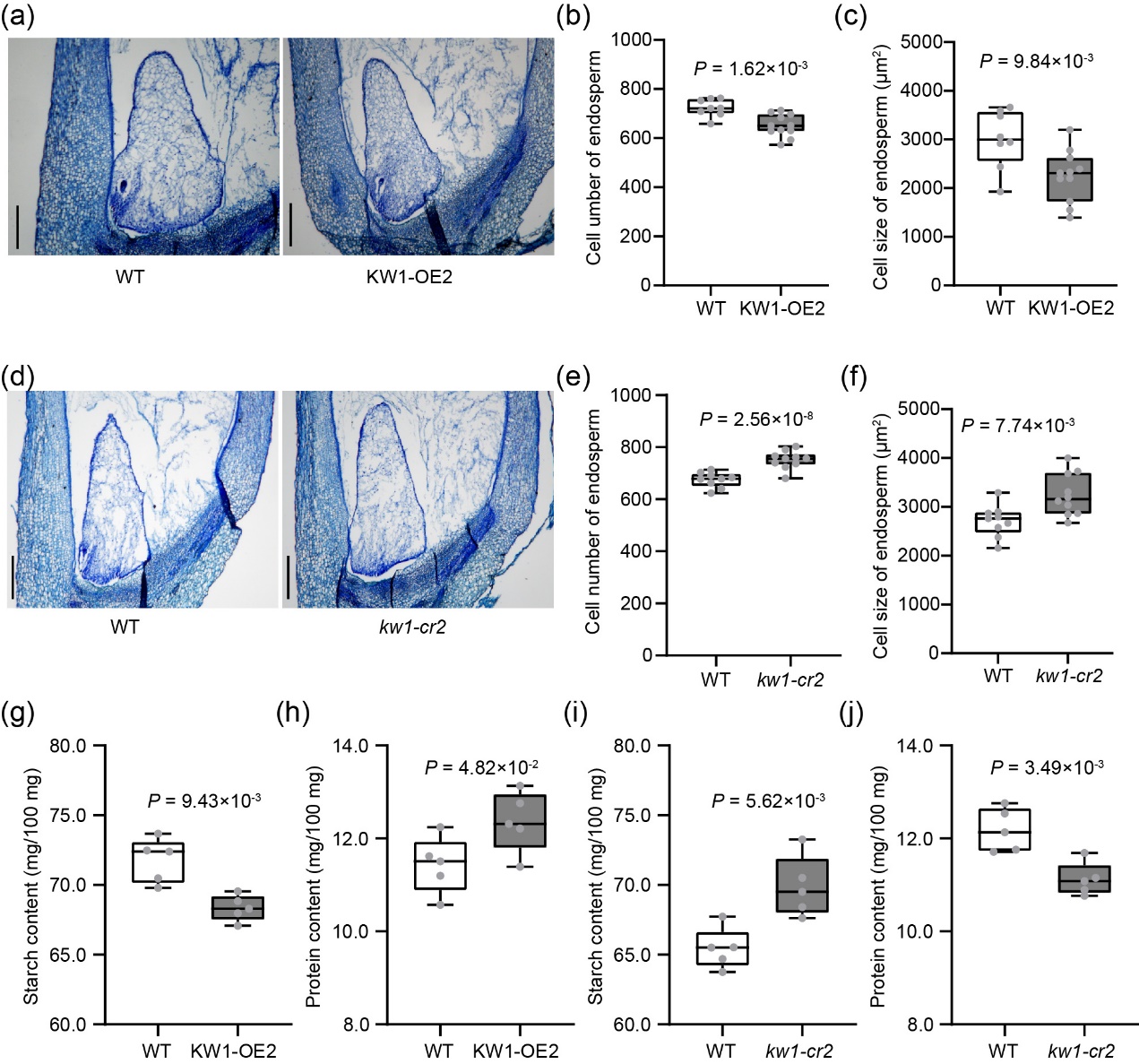


**Figure S7. Kernel phenotypes of *ZmKW1* overexpression and knockout lines.** (a) Light microscopy images of longitudinal paraffin sections of 6 DAP endosperm from the WT and KW1-OE2 lines. Scale bar, 100 μm. (b) Measurement of endosperm cell number and cell size of the wild type and KW1-OE2. Data are presented as mean values ± SD, n = 8-11 biologically independent samples. (c) Light microscopy images of longitudinal paraffin sections of 6 DAP endosperm of the WT and *kw1*-cr2 lines. Scale bar, 100 μm. (d) The measurement of endosperm cell number and cell size of the WT and *kw1*-cr2. Data are presented as mean values ± SD, n = 9-10 biologically independent samples. (e-g) Total starch contents and protein contents of the WT and KW1-OE2 (e), the WT and *kw1*-cr2 (f) mature kernels. Data are means ± SD, n = 5 biological replicates. *P* values were determined by two-tailed Student’s *t*-test.

**
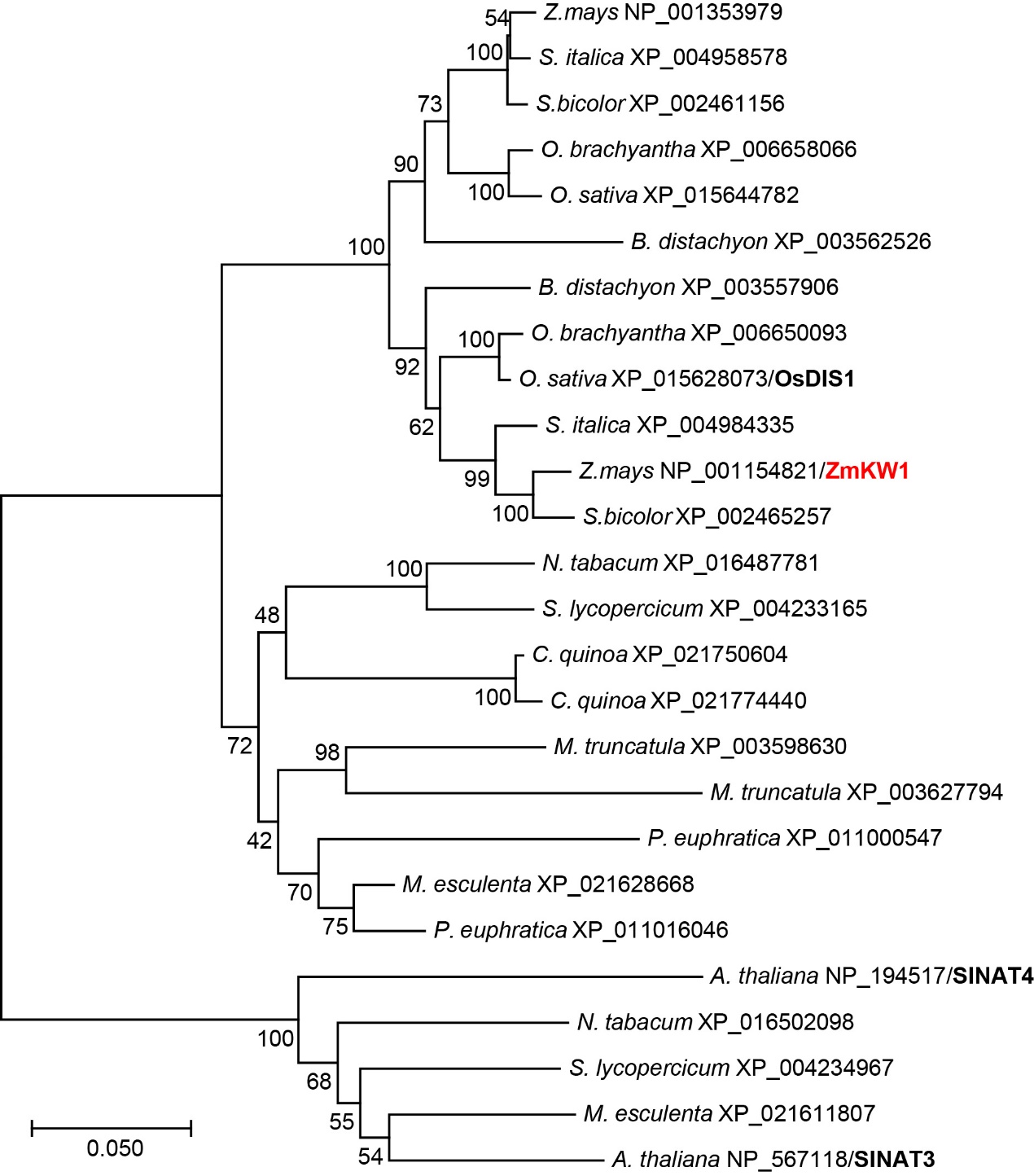
**

**Figure S8. Phylogenetic tree analysis of ZmKW1 protein containing SINA domain**. Phylogenetic relationships among SINA domain-containing proteins. Full-length amino acid sequences of SINA domain-containing paralogous and orthologous were downloaded from the NCBI and TIGR database. A phylogenetic tree was constructed using the neighbor joining algorithm in MEGA 7 software. ZmKW1 in maize, SINAT3 and SINAT4 in *Arabidopsis* are highlighted in bold.

**
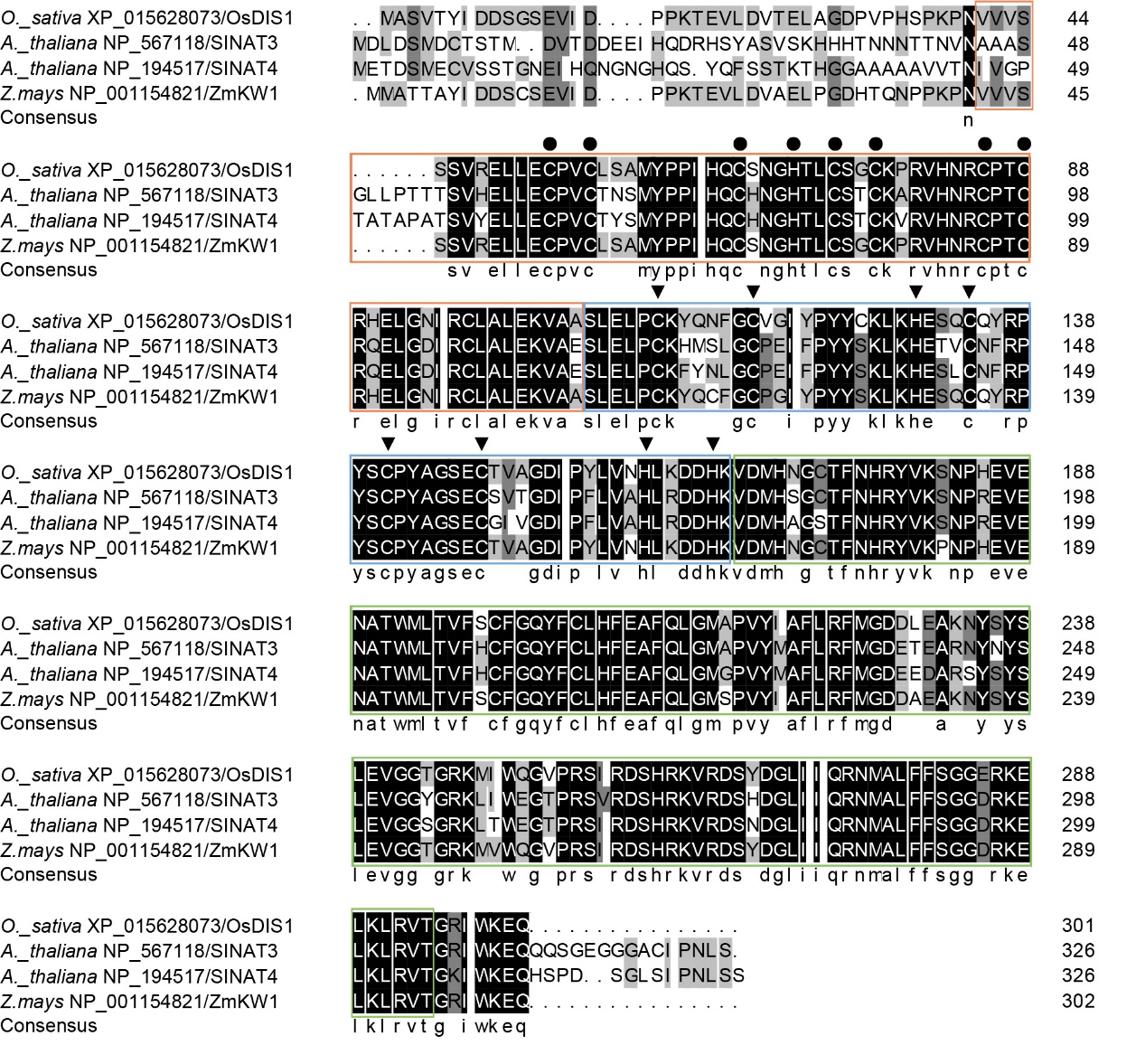
**

**Figure S9. Sequence alignment and domain structures analysis of SINA proteins.** Schematic diagram of amino acid sequences of SINA proteins were subjected to multiple sequence alignment using DNAMAN software. Three key functional domains are represented by different color blocks: the RING domain (orange), the SIAH domain (blue), and the TRAF domain (green). Circles and triangles indicate conserved cysteine and histidine residues, respectively, in the RING domain and the SIAH domain (zinc finger domain).
